# Discovery of carbamate degrading enzymes by functional metagenomics

**DOI:** 10.1371/journal.pone.0189201

**Published:** 2017-12-14

**Authors:** Lisa Ufarté, Elisabeth Laville, Sophie Duquesne, Diego Morgavi, Patrick Robe, Christophe Klopp, Angeline Rizzo, Sandra Pizzut-Serin, Gabrielle Potocki-Veronese

**Affiliations:** 1 LISBP, Université de Toulouse, CNRS, INRA, INSA, Toulouse, France; 2 UMRH, INRA, Vetagro Sup, France; 3 LibraGen S.A., Toulouse, France; 4 Plateforme Bio-informatique Toulouse Genopole, UBIA INRA, Castanet-Tolosan, France; Dong-A University, REPUBLIC OF KOREA

## Abstract

Bioremediation of pollutants is a major concern worldwide, leading to the research of new processes to break down and recycle xenobiotics and environment contaminating polymers. Among them, carbamates have a very broad spectrum of uses, such as toxinogenic pesticides or elastomers. In this study, we mined the bovine rumen microbiome for carbamate degrading enzymes. We isolated 26 hit clones exhibiting esterase activity, and were able to degrade at least one of the targeted polyurethane and pesticide carbamate compounds. The most active clone was deeply characterized. In addition to Impranil, this clone was active on Tween 20, pNP-acetate, butyrate and palmitate, and on the insecticide fenobucarb. Sequencing and sub-cloning of the best target revealed a novel carboxyl-ester hydrolase belonging to the lipolytic family IV, named CE_Ubrb. This study highlights the potential of highly diverse microbiota such as the ruminal one for the discovery of promiscuous enzymes, whose versatility could be exploited for industrial uses.

## 1. Introduction

Carbamates are *N*-substituted esters of carbamic acid. They are organic compounds with general formula R_1_-NR_2_-(C = O)O-R_3_, used in agriculture as pesticides (where R_1_ is a methyl, benzimidazole and aromatic moiety, respectively). The synthesis and commercialisation of carbamate pesticides have been in progress since the 1950’s. Nowadays, the most employed pesticides are organophosphorus compounds, carbamates and pyrethroids [1].

Carbamate pesticides have been in use in different kinds of crops all over the world. Their use increased along with organophosphorus, to replace organochlorine pesticides. However, some carbamate compounds and derivatives have an acute toxicity for mammals and aquatic organisms. Carbamates, just like the well-known toxic organophosphorus compounds, are inhibitors of acetylcholinesterase and therefore cause very similar symptoms. Some of them, containing aromatic moieties, are also suspected carcinogens and mutagens since they break down to aniline based derivatives, which were shown to be carcinogenic for humans [2,3]. For example, methyl carbamates are considered non genotoxic because of the inability of the methyl group to undergo metabolic degradation to an epoxide, while ethyl carbamates are considered as multispecies carcinogens causing malignancies in different tissues (lung, hematopoietic system, liver etc.) [4]. As such, environmental cleanup and wastewater treatment became a high priority, more than 10,000 tons of carbamates being used per year worldwide in the agriculture sector (data obtained from the Food and Agriculture Organization of the United Nations, http://faostat3.fao.org/ and from [5].

The elimination of many lipophilic environmental pollutants is based on their conversion to water soluble compounds and further microbial metabolisation. The first step in the metabolic degradation of pesticide carbamates is their hydrolysis [1,6] catalysed by carboxyl ester hydrolases (EC 3.1.1). These enzymes are part of the wide group of ester hydrolases (EC 3.1) catalysing the hydrolysis of carboxylesters (EC 3.1.1), thioesters (EC 3.1.2), phosphoric (EC 3.1.4/5/7/8) and sulfuric (EC 3.1.6) esters. Carboxylesterases from various bacteria were previously reported to hydrolyse carbamates: *Blastobacter* [7], *Arthrobacter* [8], *Pseudomonas* [9], *Achromobacter* [10] and *Micrococcus* genera [11]. Some of them display important homologies with eukaryotic carbamate-acting carboxylesterases [8,12].

Besides phytosanitary products, another type of carbamates, namely polyurethanes (PUs), causes important environmental problems. These polymers are found in many industrial products: coatings, furniture, paints, synthetic skin, construction materials, elastomers, and even implantable biomedical devices [13,14]. Gradually, they have replaced other polymers for many reasons: chlorinated rubber because of safety reasons; latex rubber because of the lower density and higher flexibility of PUs; plastics (such as PVC for automotive application for example) because of their resistance to water, oils and solvents,etc. [15]. Polyurethanes accounts for 6 to 7% of the total mass of plastic generated worldwide, with over half of the total global market for coating applications [14]. Polyurethane worldwide production thus represents 15 Mt per year. The major use of PUs in Europe is packaging, and while recycling has increased in the last few years, the most important way to dispose PUs is through landfilling [16]. Impranil®DLN, the specific PU used in this study, is a anionic aliphatic polyester-PU produced by Bayer Corporation for use in textile, leather and aircraft fabric coating [14].

PU biodegradation results from the activity of two main classes of enzymes [15,17]: esterases (such as cholesterol esterases) and proteases (such as papain) that were shown to hydrolyse carbaryl and 4-nitrophenyl *N*-methyl-carbamates [18–20]. Other kinds of enzymes such as ureases [21,22], urethanases [23,24], along with enzymes named impranilases, were shown to degrade PUs. Moreover, PU degrading bacteria are numerous, mostly from the phyla Firmicutes, Actinobacteria, and Proteobacteria, although the enzymes involved in these processes have not all been identified to date [20]. It was shown, however, that PUs are barely used as carbon or nitrogen sources for microorganisms in strict anaerobic conditions [25]. On the other hand, in aerobic conditions, only one bacterial strain, assigned to the *Comamonas* genus, has been shown to use polyester PU as carbon and nitrogen source [26].

Despite these advances in the understanding of carbamate enzymatic degradation, there are still very few biological routes identified for the breakdown and recycling of carbamate-based xenobiotics and polymers. In particular, the carbamate degrading potential of the uncultivated bacterial fraction, which represents between 70 and 99% of most microbial ecosystems, is underexplored. Only one study indeed allowed the discovery of an esterase from an uncultivated soil bacterium [27], capable of degrading poly(diethylene glycol adipate, a component of polyester polyurethanes. In addition, to our knowledge, no metagenome was mined for activities of cleavage of the carbamate linkage of pesticides [28].

The bovine rumen metagenome was chosen for this study because of its great functional and microbial diversity [29], and because of its richness in esterases, including carbohydrate-esterases [29,30] and lipases [31,32]. In this dense ecosystem, these enzymes are involved both in the metabolisation of lipids and of plant cell wall components, since carbohydrate-esterases catalyze the de-O or -N-acylation of substituted hemicelulloses and pectins [33].

Here we report the discovery, by using a multi-step activity-based metagenomics strategy targeting the bovine rumen microbiome, of new esterases capable of hydrolysing two different structures of carbamates, more specifically Impranil and fenobucarb.

## 2. Results and discussion

### 2.1. Screening of the bovine rumen metagenomic library

The bovine rumen metagenomic library consisted of 19,968 *Escherichia coli* fosmid clones, each clone comprising a 30–40 kb DNA insert. The library covers around 700 Mbp of metagenomic DNA, which corresponds to the equivalent of more than one hundred bacterial genomes.

The library was first screened for the presence of chosen enzymatic activities: both esterases and proteases were searched for, since they were previously reported to cleave the carbamate linkage. The ability of a clone to degrade Tween 20 (polyethylene glycol sorbitan monolaurate, an ester with C12 chain length), revealed the presence of esterases and lipases, also renamed non lipolytic esterases and lipolytic esterases, respectively, according to the classification of Ben Ali et al. [34], whereas the ability to degrade AZCL-casein indicated protease activity.

The screen on Tween 20 resulted in 26 positive clones, corresponding to a hit yield of 0.13%. This value is in the same range of those obtained from other activity-based metagenomics studies targeted on the bovine rumen microbiome. Indeed, [35] found 11 esterases from the rumen metagenome (hit yield 0.08%) by using α-naphtyl acetate, a short chain C2 naphtyl ester, as the screening substrate. In another study, [32] found 9 positive clones (hit yield 0.04%) on tributyrin, a C4 chain triglyceride. The high hit yield obtained in the present study indicates that Tween20 is an appropriate substrate to explore the functional diversity of bacterial carboxylesterases.

The screen on AZCL-casein did not allow the detection of any positive clone, contrarily to what we previously observed in the same screening conditions with other metagenomic libraries issued from various composts (unpublished results). A secondary screening of the 26 hit clones was performed on two different kinds of substrates: Impranil, a commercial polyester polyurethane, and *p*NP chromogenic esters of various chain lengths (C2, C4 and C16) to discriminate and quantify lipolytic versus non lipolytic carboxylesterase activities.

Impranil degradation was screened by using both positive selection on mineral medium containing the PU as sole carbon source, and by searching for clones, grown on rich medium, showing a polymer degradation halo [19]. Among the 26 clones obtained in total after primary screening, 22 positive clones were obtained on the rich medium supplemented with Impranil. Thirteen of them were also positive on the mineral medium containing Impranil as sole carbon source. They correspond to those harbouring the largest Impranil degradation halo on the rich medium, due either to high recombinant gene expression and/or to high enzyme efficiency and/or even to secretion which may happen, even rarely, in *E*. *coli*. Furthermore, of the 22 clones active on Impranil, 5 were not active on the chromogenic esters, 2 were active on *p*NPA only, 3 on *p*NPB only, 10 on both *p*NPA and *p*NPB, and 2 on *p*NPA, *p*NPB and *p*NPP, the latter substrate being specific to lipolytic carboxylesterases [36]. Of the remaining 4 clones that were not active on Impranil, 2 were not active on the chromogenic esters, 1 was active on both *p*NPA and *p*NPB, and 1 on *p*NPA, *p*NPB and *p*NPP.

These results highlight the diversity of substrate specificities of ruminal bacterial esterases. Screening using different substrates allowed the clustering of hit clones based on their functional diversity (S1 Table). From the 22 clones highly active on Impranil, 8 were chosen for further sequencing in order to identify novel enzymes active on carbamates. In order to explore the largest sequence and functional diversity as possible, the clones to be sequenced were selected because i) they were all able to degrade Impranil, ii) they belonged to various functional clusters, especially to those which contain the highest number of clones, or the most multispecific clones. Among them, clone 44I12 was one of the most efficient to break down Impranil, as it produced the largest degradation halos. This clone was active on Impranil with both assays. This characteristic is particularly important since it shows the ability of the clone to use Impranil as a carbon source, since recent studies have spread some doubts on the specificity of the screening methods based on the observation of a degradation halo on solid media for the discovery of Impranil degrading enzymes and strains [14]. Clone 44I12 was also active against all 3 tested *p*NP-esters and it was selected for in depth characterization of its activity on carbamates of various structures (S1 Fig).

### 2.2. Polyurethane degradation

**Fig 1:**
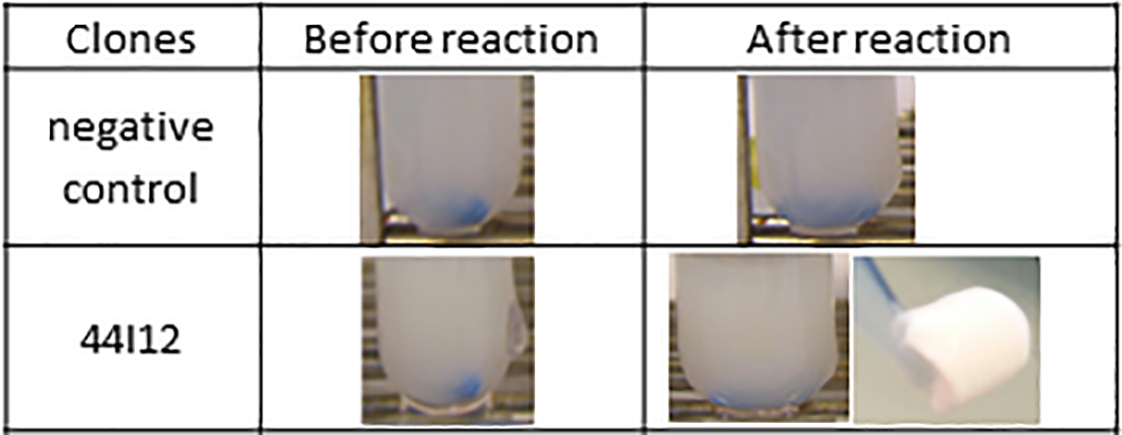
Reaction media of 44I12 cytoplasmic extract incubated with Impranil at 30°C and pH 7.0 during 24h. Negative control: *E*. *coli* host strain Epi100 transformed with the empty pCC1FOS fosmid. For 44I12, the reaction medium, in and out of the tube, is showed, since a solid aggregate could be obtained.

In order to further investigate the action of clone 44I12 on polyurethane, the concentrated cytoplasmic extract was incubated in the presence of Impranil. After one day of reaction at 30°C and pH 7.0, the medium became compact and viscous (Fig 1), and finally a solid aggregate was obtained. We compared these observations with those of [14], who used various commercial enzymes to degrade Impranil: an esterase from *Pseudomonas fluorescens* with a broad substrate specificity (from lysophospholipase to amidase activity), Savinase™, a commercial serine-type protease originally produced by *Bacillus* sp., and a triacylglycerol lipase from *Pseudomonas* sp.. Biffinger et al. [14] observed that 2 days incubation at 30°C of Impranil with the esterase and lipase resulted in a decrease of the reaction medium opacity without aggregate formation, whereas Impranil aggregated when incubated with protease. In the present work, however, this effect on Impranil could not be attributed to proteolytic activity, since no protease activity was detected by testing clone 44I12 on Hide Remazol Blue, AZO- and AZCL-casein (data not shown).

**Fig 2:**
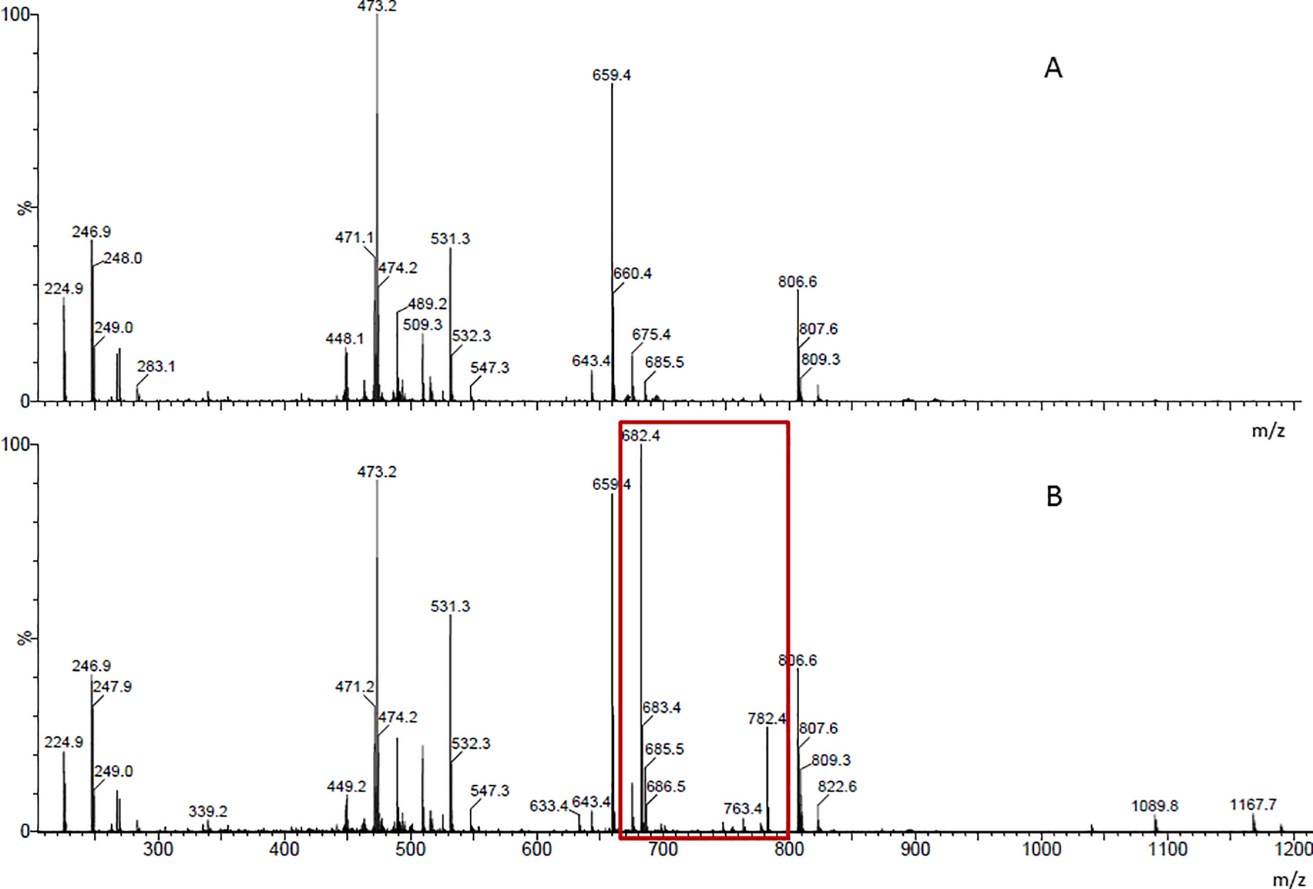
**MALDI-TOF spectra of the reaction medium containing Impranil and the enzymatic extract of clone 44I12 at the beginning (A) and after 24h (B) of reaction.** The red rectangle shows the new peaks observed after reaction.

In order to analyse the molecular mass distribution of the Impranil substrate and reaction products, MALDI-TOF analyses were performed on reaction media before and after reaction. The spectra obtained with clone 44I12 (Fig 2) presented significant differences before and after reaction. A different molar mass distribution was observed, with the appearance of peaks at m/z 682.4, 683.4 and 782.4 after incubation. This was not the case when Impranil was incubated with the negative biotic control, ie the *E*. *coli* strain Epi100 carrying the empty pCC1FOS fosmid (S2 Fig). The spectra are in this case very similar before and after incubation. In addition, the spectrum obtained in the same analysis conditions with Impranil resuspended in THF (S2 Fig) indicates a regular molar mass distribution which is very different than that obtained with the biotic samples, which logically contain a lot of impurities. Unfortunately, it is still impossible to relate the molar mass distribution to a specific polymeric structure, since there is no known structure of Impranil [19], even if a hypothetical one has been proposed by Biffinger *et al*. [14]. According to this hypothetical structure, the predominant new peaks generated by clone 44I12 could correspond to the following degradation products: the one at m/z 682.4-is the closest in size to C_36_H_68_O_8_N_2_ with an atom of Na, with 683.4 a possible isotope; the one at 782.4 could be the motif C_36_H_66_O_8_N_2_ with an atom of I but nothing seems to match with only additions of Na^+^ or I^-^. These peaks were not observed on the spectra obtained after incubation of clone 44I12 with two other polyurethanes (namely poly[4-(2,2-dicyanovinyl)-N-bis(hydroxyethyl)aniline-alt-(4,4’-methylenebis(phenylisocyanate)))]urethane and poly[4-(2,2-dicyanovinyl)-N-bis(hydroxyethyl)aniline-alt-(isophroronediisocyanate)]urethane), on which 44I12 was not active (S3 Fig). This indicates that the peaks at m/z 682.4, 683.4 and 782.4 are specific to clone 44I12 action on Impranil, and not to a modification of a compound contained in the cytoplasmic extract. These results constitute a definite proof, along with those of screening, that the enzymes produced by clone 44I12 impact the structure of the commercial polyurethane Impranil.

### 2.3. Pesticide carbamate degradation

Clone 44I12 was then tested on fenobucarb, fenoxycarb, and prosulfocarb, three synthetic carbamate pesticides of known structures (S1 Fig). Fenobucarb is an insecticide with a high potential for bio-concentration, used in many rice growing countries worldwide. Fenoxycarb is also an insecticide, but of which bioaccumulation potential is moderate. Finally, prosulfocarb is an herbicide which is very persistent in water and sediments [37].

**Fig 3:**
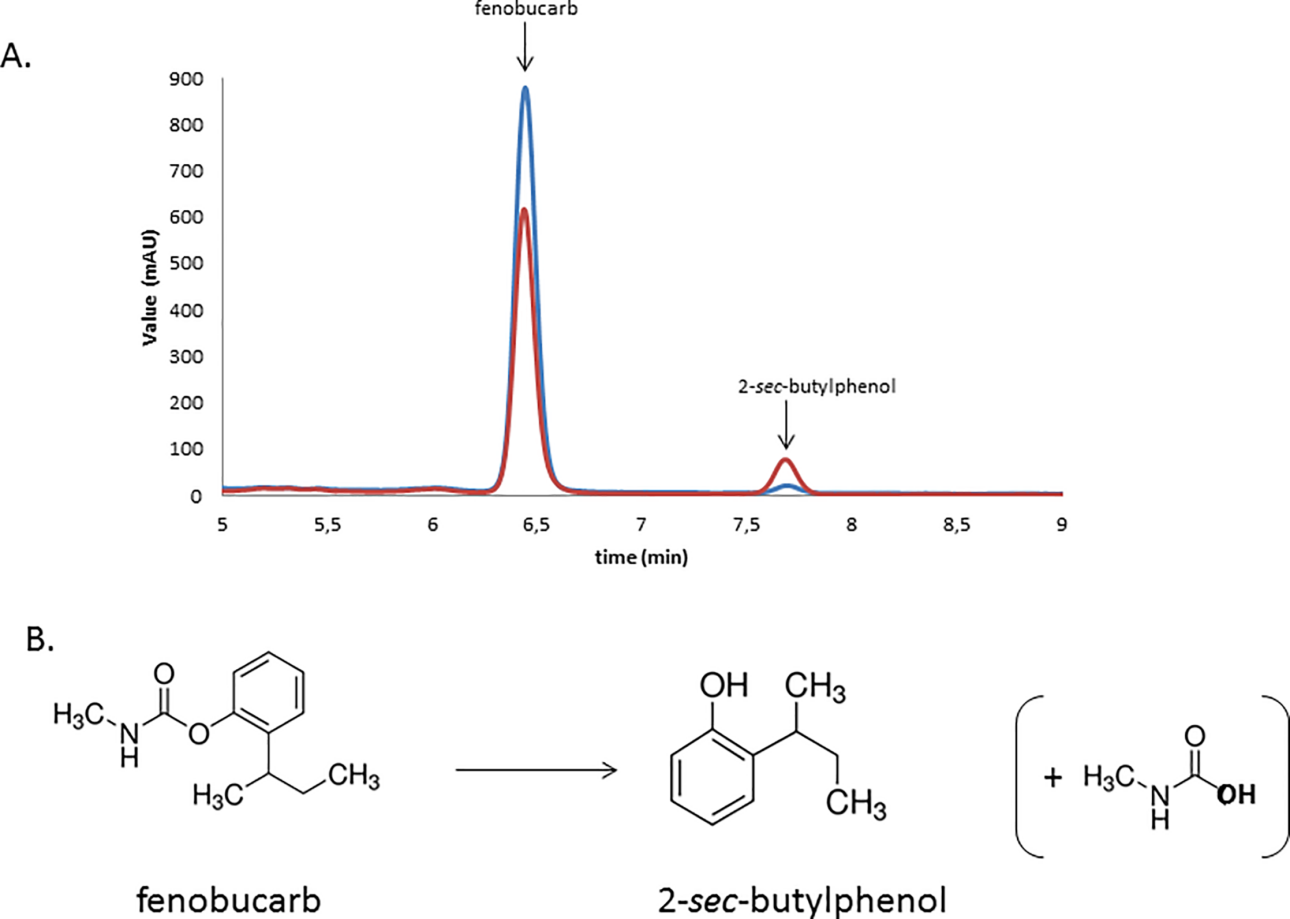
A. HPLC analysis from the reaction of fenobucarb with 44I12 enzymatic extract at the beginning of the reaction (blue) and after 24 h of reaction (red). B. Convertion of fenobucarb to 2–sec-butylphenol.

The cytoplasmic extract of clone 44I12 was incubated during three days at 30°C with these 3 compounds independently, and reaction media were analysed by LC-MS. Nothing significant was observed for the reactions on fenoxycarb and prosulfocarb, while the UV profile of the reaction medium with fenobucarb was significantly changed after reaction (Fig 3). The fenobucarb peak at retention time 6.4 min decreased by 33 ± 4% after reaction (against only by 3% after incubation with the cellular extracts of the *E*. *coli* strain Epi100 carrying the empty pCC1FOS fosmid, as shown in S4 Fig), at the expense of a second peak with a retention time of 7.7 min. This peak, which is present in very little amount on the commercial fenobucarb UV profile, corresponds to the degradation product 2-*sec*-butylphenol [38], which was checked with a commercial standard. Moreover, mass spectrum analysis allowed us to confirm that the peak at 6.4 min corresponds to fenobucarb (m/z 207) while the peak at 7.7 min presents MS peaks at masses (m/z 150 and 121) that correspond to 2-*sec*-butylphenol. Reference masses were taken from the work of [3], who studied fenobucarb degradation using membrane anodic Fenton treatment. These results demonstrate that clone 44I12 is able to degrade fenobucarb by cleaving the ester link, releasing a product (2-*sec*-butylphenol) which is less toxic than fenobucarb itself [39]. Nevertheless, clone 44I12 is unable to degrade fenoxycarb and prosulfocarb. This observation was already noted by Kim *et al*. [38], who discovered bacterial strains from the genera *Sphingobium* and *Novosphingobium*, which are able to degrade fenobucarb and carbaryl, but not other carbamates like fenoxycarb.

### 2.4. Sequence analysis

The metagenomic DNA inserts of the 8 hit clones chosen as previously described in this paper were sequenced. For each clone, the largest contig ranged in size between 21 and 38 kbp and had between 21 and 35 predicted ORFs. Two clones present a high redundancy (25I16 and 50E19), their sequence overlapping between ORF 5 and ORF 29 of 25I16 and ORF1 and ORF25 of 50E19. Such sequence redundancy frequently occurs between sequences issued from activity-based screening approaches due to the high selection pressure imposed by this strategy [40,41].

The taxonomic annotation of the largest contigs (Table 1) indicates that all sequences are issued from bacteria of which the genome has not been sequenced yet. Contigs were all assigned to Firmicutes, which is the dominant phylum in the bovine rumen microbiota [42].

**Table 1:** ORFs of interest from the sequenced clones, annotated as putative esterases or proteases.

Clone ID and accession number	Substrates	Taxonomic annotation(Phylum/Class/Genus)	ORFs of interest (accession number)	RAST annotation	Best BLAST hit against the NCBI NR database (accession number)Sequence coverage, sequence identity	Best BLAST hit with a functional annotation
13M17LT674547	Tween 20, Impranil (both media)	Firmicutes/Clostridia/ Thermacetogenium	SIP63269.1	Hypothetical protein	hypothetical protein [Ruminococcus callidus] (WP_021683508.1) Cov. 99%, Id. 53%	metallopeptidase [Anaerostipes hadrus] (WP_044924108.1) Cov. 98%, Id. 52%
			SIP63282.1	Esterase/lipase	hypothetical protein [Butyrivibrio fibrisolvens] (WP_051216853.1) Cov. 100%, Id. 73%	esterase [Clostridiales bacterium S7-1-4] (WP_034547640.1) Cov. 99%, Id. 39%
25I16LT674546	Tween 20, Impranil (both media),*p*NPA, *p*NPB	Firmicutes/Clostridia/Clostridium	SIP63246.1	Hypothetical protein	putative transglutaminase/protease [uncultured bacterium Contig1767] (AHF23837.1) Cov. 100%, Id. 100%	putative transglutaminase/protease [uncultured bacterium Contig1767] (AHF23837.1) Cov. 100%, Id. 100%
			SIP63257.1	Putative esterase	putative esterase [uncultured bacterium Contig1767] (AHF23826.1) Cov. 100%, Id. 99%	putative esterase [uncultured bacterium Contig1767] (AHF23826.1) Cov. 100%, Id. 99%
			SIP63261.1*	Hypothetical protein	hypothetical protein [Lachnospiraceae bacterium C6A11] (WP_035628054.1) Cov. 99%, Id. 46%	diguanylate phosphodiesterase [Butyrivibrio proteoclasticus] (WP_013280495.1) Cov. 99%, Id. 28%
29D17LT674545	Tween 20, Impranil(both media),*p*NPA, *p*NPB	Firmicutes	SIP63220.1	Esterase/lipase	esterase [Lactobacillus reuteri] (WP_003670556.1) Cov. 97%, Id. 50%	esterase [Lactobacillus reuteri] (WP_003670556.1) Cov. 97%, Id. 50%
			SIP63224.1	Hypothetical protein	hypothetical protein [Erysipelotrichaceae bacterium NK3D112] (WP_051665678.1) Cov. 97%, Id. 52%	peptidase family M20/M25/M40 [Firmicutes bacterium CAG:129] (CCZ45637.1) Cov. 96%, Id. 49%
37F15LT674544	Tween 20, Impranil (rich medium),*p*NPA, *p*NPB	Firmicutes/Clostridia/ Subdoligranulum	SIP63187.1	putative alpha-dextrin endo-1, 6-alpha-glucosidase	carbohydrate esterase [Streptococcus henryi] (WP_026183046.1) Cov. 100%, Id. 49%	carbohydrate esterase [Streptococcus henryi] (WP_026183046.1) Cov. 100%, Id. 49%
			SIP63194.1	Esterase/lipase-like protein	esterase [Lactobacillus reuteri] (WP_003666275.1) Cov. 96%, Id. 50%	esterase [Lactobacillus reuteri] (WP_003666275.1) Cov. 96%, Id. 50%
39F1LT674543	Tween 20, Impranil (both media),*p*NPA, *p*NPB	Firmicutes/ Erysipelotrichia/ Holdemania	SIP63165.1	FIG004556: membrane metalloprotease	m50 family membrane metalloprotease [Faecalibacterium sp. CAG:74] (CDE51946.1) Cov. 99%, Id. 46%	m50 family membrane metalloprotease [Faecalibacterium sp. CAG:74] (CDE51946.1) Cov. 99%, Id. 46%
			SIP63179.1	Predicted metal-dependant phosphoesterase	PHP domain-containing protein [Clostridiales bacterium], WP_084096767.1, Cov. 97%, Id.54%	predicted metal-dependent phosphoesterases (PHP family) [Ruminococcus sp. CAG:379], CDD54255.1, Cov.96%, Id. 46%
44I12LT674542	Tween 20, Impranil (both media),*p*NPA, *p*NPB, *p*NPP	Firmicutes/Bacili/Thermobacillus	SIP63154.1*CE_Ubrb	probable lipase/esterase	hypothetical protein [[Clostridium] stercorarium] (WP_015360182.1)Cov. 95%, Id. 46%	esterase [Lactobacillus vaginalis] (WP_003717503.1) Cov. 95%, Id. 43%
50E19 LT674541	Tween 20, Impranil (both media), *p*NPA, *p*NPB	Firmicutes/Clostridia/Clostridium	SIP63132.1	Putative esterase	putative esterase [uncultured bacterium Contig1767] (AHF23826.1) Cov. 100%, Id. 99%	putative esterase [uncultured bacterium Contig1767] (AHF23826.1) Cov. 100%, Id. 99%
			SIP63121.1	Hypothetical protein	putative transglutaminase/protease [uncultured bacterium Contig1767] (AHF23837.1) Cov. 100%, Id. 100%	putative transglutaminase/protease [uncultured bacterium Contig1767] (AHF23837.1) Cov. 100%, Id. 100%
			SIP63136.1*	Hypothetical protein	hypothetical protein [Lachnospiraceae bacterium C6A11] (WP_035628054.1) Cov. 99%, Id. 46%	diguanylate phosphodiesterase [Butyrivibrio proteoclasticus] (WP_013280495.1) Cov. 99%, Id. 28%
50I3LT674540	Tween 20, Impranil (rich medium), *p*NPA, *p*NPB	Firmicutes/Bacili/Bacillus	SIP63105.1*	N-acetylglucosamine-6-phosphate deacetylase (EC 3.5.1.25)	n-acetylglucosamine-6-phosphate deacetylase [Ruminococcus sp. CAG:382] (CDD02602.1) Cov. 99%, Id. 55%	n-acetylglucosamine-6-phosphate deacetylase [Ruminococcus sp. CAG:382] (CDD02602.1) Cov. 99%, Id. 55%
			SIP63109.1	Putative esterase	hypothetical protein [Hungatella hathewayi] (WP_006780815.1)Cov. 96%, Id. 57%	tributyrin esterase [Clostridiales bacterium VE202-28] (WP_025484029.1) Cov. 96%, Id. 56%

*ORFs encoding putative proteins of theorical molecular weight corresponding to overproduced proteins in fosmid clones, as observed by SDS-PAGE analysis of cytoplasmic extracts (S6 Fig)

**Fig 4:**
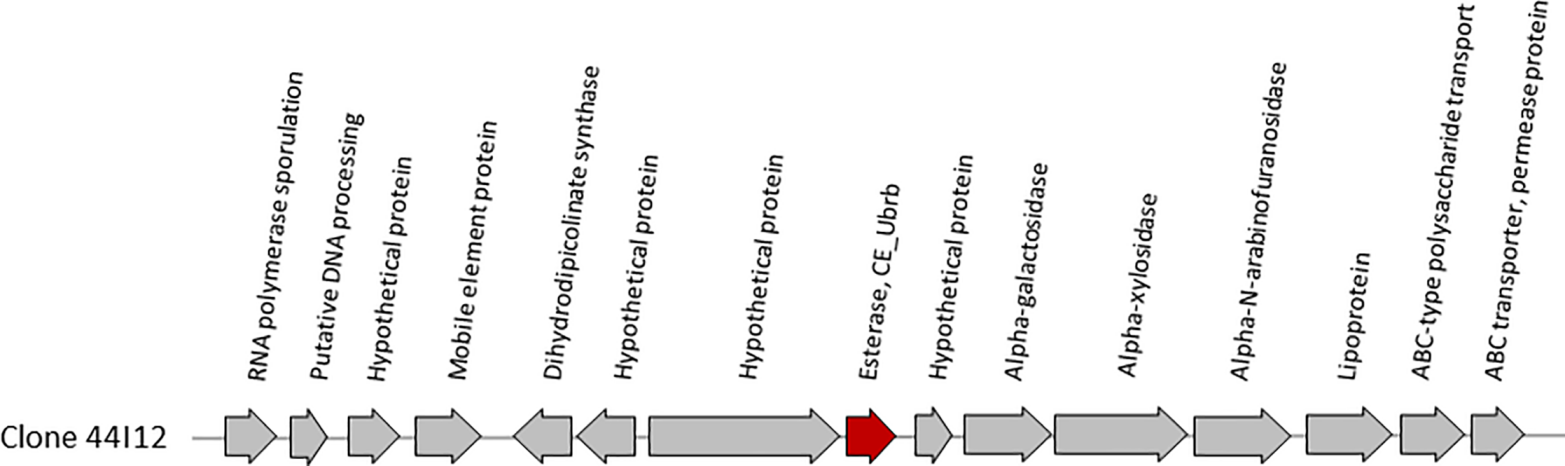
Graphical representation of the metagenomic sequence of clone 44I12, and RAST annotation of the identified ORFs. Red arrow: CE_Ubrb encoding gene.

The clone sequences were then mined for genes annotated as encoding putative esterases, ureases or proteases that could be responsible for the screened activities. We have not detected any potential target in small contigs, which contained a maximum of 1 or 2 ORFs. The RAST annotation of the largest contigs allowed us to identify at least one gene predicted to encode an esterase or a protease for each clone. These annotations were confirmed by BLASTP comparison of the ORF sequences to the NR database. In addition, the BLASTP analysis revealed other genes encoding putative esterases or proteases that were annotated by RAST as ‘hypothetical proteins’. Among the 8 sequenced hit clones, 5 contain at least two genes encoding putative enzymes that may act synergistically on the targeted substrates. All together, we found 9 different putative esterase and 6 putative protease sequences (Table 1, Fig 4 and S5 Fig). The fact that no protease activity was found after primary and secondary screening indicates either that the putative protease encoding genes were not properly expressed in *E*. *coli*, or, if well expressed, that these enzymes are unable to break down the particular structure of the substrates used in this study.

According to Gabor *et al*., [43], *E*. *coli* is predicted to readily express around 40% of environmental genes, with strong variations depending on source organisms. And as previously shown, metagenomic genes cloned into fosmids are spuriously transcribed in *E*. *coli* thanks to *E*. *coli* promoter sequences that are randomly distributed along the metagenomic DNA insert [44]. Here, expression of the genes annotated as putative esterases or proteases was checked by SDS-PAGE analysis of the cytoplasmic extracts of the 8 sequenced clones. For all clones, we visualized one or several proteins overproduced compared to the negative control. For clones 25I16, 44I12, 50E19 and 50I3, the size of one of these proteins corresponds to that of the putative esterases identified by functional annotation (S6 Fig, Table 1). Without being a proof of their function, these results strengthen the value of the functional annotation. They also demonstrate again the potential of *E*. *coli* to express metagenomic genes issued from bacteria that are very distant from Proteobacteria.

The sequence of clone 44I12 was further analysed. It contains no ORF annotated as protease, and only one ORF (SIP63154.1) annotated by the RAST server as a probable lipase/esterase. The BLASTP analysis of all the other hypothetical proteins did not allow us to identify another putative esterase. We therefore hypothesized that this gene was responsible for the activities of clone 44I12. In order to prove it, we subcloned this gene individually in the overexpression vector pET55. The predicted molecular weight of the recombinant protein fused to a N-terminal Strep-tag and to a C-terminal (His)_6_ tag is 36,4 kDa, against 32,5 kDa for the native protein produced by the 44I12 fosmid clone. The SDS-PAGE analysis of the cytoplasmic proteins of the E. coli BL21star (DE3) strain transformed with this plasmid highlighted the overproduction of the full-size recombinant protein, at a slightly higher level than that produced by the 44I12 fosmid clone (S7 Fig). Moreover, in agreement with these results, the level of esterase activity of the plasmid clone on *p*NP-esters (5.26, 2.45 and 0.032 U/mL of cytoplasmic extract for *p*NPA, *p*NPB and *p*NPP, respectively) was on average 1.3 times higher than that produced in the same conditions by the fosmid clone. These results confirm that the protein with accession number SIP63154.1 is an esterase. We named it CE_Ubrb (“carboxyl-esterase from an uncultured bovine ruminal bacterium”). Its best characterized homolog is the esterase LC-Est2 from an uncultured bacterium, discovered from a leaf-branch compost metagenome [45]. LC-Est2 shares 32% identity on 94% of sequence length with CE_Ubrb, and is active on tributyrin, a substrate specific to esterases and lipases. This is in accordance with our results, CE_Ubrb being active on all *p*NP esters tested. LC-Est2 has never been tested on carbamates, to our knowledge.

Six other characterised enzymes were found among the 5,662 sequences of the NCBI NR database presenting more than 25% identity with CE_Ubrb. These characterised enzymes are named lipases/esterases, or lipolytic enzymes. All of them are part of the serine hydrolase family, containing the GXSXG consensus sequence, and take part in prokaryotic and eukaryotic lipolysis (Table 2).

**Table 2:** Characteristics of the characterised enzymes with more than 25% identity with CE_Ubrb.

Accession number	Annotation and substrate specificity	Sequence identity	Coverage	Taxonomic origin	Reference
**AIT56388**	esterase LC-Est2 : tributyrin	32%	95%	Uncultured bacterium	[45]
**AFG17170**	EstJ : tributyrin, *p*-nitrophenyl esters (C2-4-6-8-10-12-14)	29%	89%	Uncultured bacterium	[46]
**ACF04196**	lipase/esterase : tributyrin,*p*-nitrophenylbutyrate/ caprylate/laurate	28%	91%	Uncultured bacterium	[47]
**ACL67843**	lipolytic enzyme : tributyrin, *p*-nitrophenylbutyrate	27%	87%	Uncultured bacterium	[48]
**ABQ11272**	lipase/esterase : Tributyrin, *p*-nitrophenylbutyrate	29%	63%	Uncultured bacterium	[49]
**AAS77245**	lipase/esterase : Tributyrin, *p*-nitrophenylbutyrate	27%	65%	Uncultured bacterium	[50]
**AAS77242**	lipase/esterase : Tributyrin, *p*-nitrophenylbutyrate	26%	63%	Uncultured bacterium	[50]

**Fig 5:**
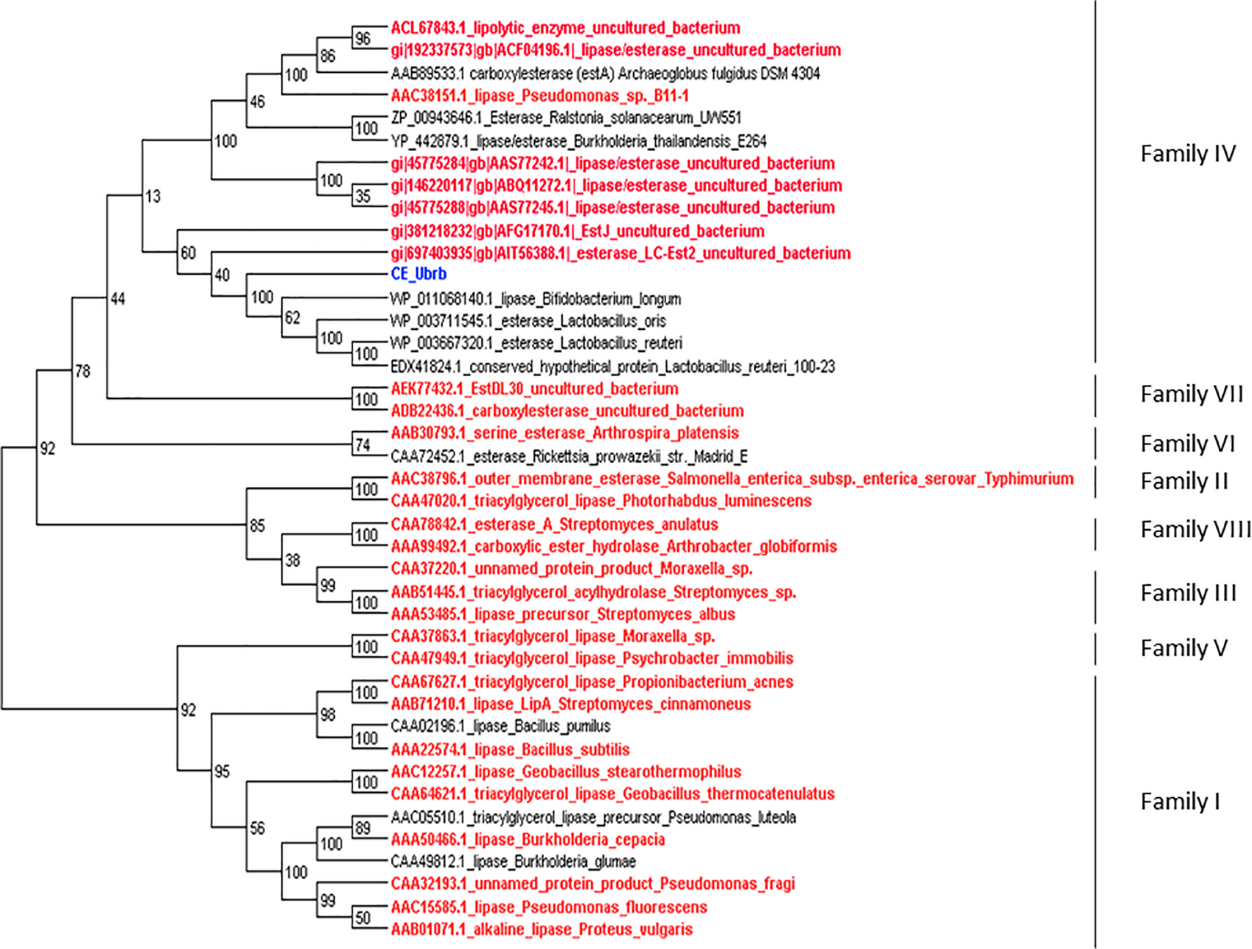
Phylogenetic tree of sequences characteristic of the first 8 lipolytic families described by Arpigny et Jaeger [51], along with the characterized CE_Ubrb blast hits against the NCBI-NR database and the first blast hits against the ESTHER database. In red: biochemically characterized enzymes.

According to Arpigny et Jaeger [51], bacterial lipolytic enzymes can be classified in eight families, depending on the conserved sequence motif and their biological properties. Against the ESTHER database [52], which clusters in 4 blocks more than 30.000 manually curated proteins displaying an α/β-hydrolase fold, the best CE_Ubrb BLAST hit was found to be *Lactobacillus reuteri* 100–23 HSL (EDX41824.1), sharing 44% of identity over 99% coverage. This putative enzyme, which is still not characterised, is a member of the HSL-like family from block H, which corresponds to family IV of the classification of lipolytic enzymes [51]. This family is composed of many esterases from distantly related prokaryotes [53,54]. These enzymes are remarkably similar to mammalian hormone-sensitive lipases (HSL), containing the serine, aspartic acid and histidine catalytic residues, along with a G that is involved in hydrogen bonding interaction, stabilizing an oxyanion hole formed during enzymatic reaction and promoting the catalysis [55]. All these residues are conserved in the CE_Ubrb sequence (S8 Fig). Bacterial HSL supposedly show activity only towards short fatty acid chain length substrates (tributyrin, vinyl propionate) [56] compared to mammalian HSL that have a broad substrate specificity (olive oil, short chain esters, trioctanoin, etc.) The CE_Ubrb activity profile shows a closer similarity to mammalian HSL than to bacterial ones, our target being active on short chain esters but also on Impranil and its long motif. Finally, the ESTHER database also mentions the affiliation of these HSL-like family to the block H, containing, among others, plant carboxylesterases and bacterial tannases. It also contains kynurenine-formamidases, a pattern found in the sequence of CE_Ubrb using the InterProScan software. According to the tree we created with sequences from the first 8 lipolytic families described in Arpigny et Jaeger (Fig 5), it is also clear that the CE_Ubrb sequence clusters with all family IV members, in particular the 6 of the 7 characterized best blast hits listed in Table 2. However, surprisingly, Lc-Est2 (which, according to Okano et al. [45], would belong to the family I-6) also clusters in the family IV branch, even if it is more distantly related to family IV members than CE_Ubrb itself. Anyway, no significant homology can be found between CE_Ubrb and family VII members, while all other carbamate degrading enzymes identified to date exclusively belong to family VII [55].

## 3. Material and methods

### 3.1. Chemicals

Impranil DLN was supplied by Bayer Corporation (Germany). Poly[4-(2,2-dicyanovinyl)-N-bis(hydroxyethyl)aniline-alt-(4,4’-methylenebis(phenylisocyanate)))]urethane and poly[4-(2,2-dicyanovinyl)-N-bis(hydroxyethyl)aniline-alt-(isophroronediisocyanate)]urethane were purchased from Sigma Aldrich (France).

Tween 20, Pefabloc, *para*-nitrophenyl acetate (*p*NPA), *para*-nitrophenyl butyrate (*p*NPB), *para*-nitrophenyl palmitate (*p*NPP), *para*-nitrophenol (*p*NP), tetrahydrofuran (THF), 2-*sec*-butylphenol, (2-Butan-2-ylphenyl), N-methylcarbamate (fenobucarb), Ethyl 2-(4-phenoxyphenoxy)ethylcarbamate (fenoxycarb) and S-Benzyl dipropylthiocarbamate (prosulfocarb) were purchased from Sigma Aldrich (France).

AZCL-xylan (XYL), AZCL-casein (CAS), AZCL-Barley β-glucan (BGLU), AZO-CM Cellulose (CMC), AZO-Carob Galactomannan (GM) were purchased from Megazyme (Ireland).

Lysonase™ Bioprocessing Reagent was purchased from Novagen (France).

### 3.2. Metagenomic DNA sampling and library construction

Rumen contents were obtained from two non-producing, rumen-cannulated Holstein dairy cows. Cows were housed at the Herbipole experimental unit (INRA, Theix). Procedures on animals complied to national standards fixed by the legislation on animal care (Certificate of Authorization to Experiment on Living Animals, No. 004495, Ministry of Agriculture, France). They were adapted during 7 weeks to a diet of 80% wheat straw, 12% concentrate and 8% beetroot molasses. Feeding was *ad libitum* once a day in the morning. Rumen contents were taken before feeding from various parts of the rumen and manually homogenized.

The metagenomic library was constructed from the mix of rumen samples from both cows. Enriched bacterial fractions were recovered separately from the two rumen samples by a density gradient technique using Nycodenz as previously described [57]. The cell pellets were suspended in a 50 mM Tris (pH 8), 100 mM EDTA buffer, mixed in equal weight parts and then incorporated in low-melt-point agarose before a gentle enzymatic lysis as previously described [40]. Fragments sizing between 30 and 40 kb were isolated and cloned into pCC1FOS fosmid (Epicentre Technologies). EPI100 E. *coli* cells were then transfected to obtain a library of 19,968 clones from the rumen samples. Recombinant clones were transferred to 384-well microtiter plates containing Luria Bertani (LB) medium, supplemented with 12.5 mg/L chloramphenicol and 8% (w/v) glycerol. After 22 hours of growth at 37°C without shaking, the plates were stored at -80°C.

### 3.3. Primary high-throughput screening of the metagenomic library

The library was gridded on 22 cm x 22 cm trays containing solid agar medium supplemented with 12.5 mg/L chloramphenicol, using an automated microplate gridder (K2, KBiosystem, Basildon, UK), which allowed to perform 140,000 assays in 2 days.

Carboxylesterase screening medium was constituted by solid LB medium supplemented with 12.5 mg/L chloramphenicol and with 1% (w/v) final concentration of Tween 20. The assay plates were incubated during 3 days at 37°C until apparition of a powdery halo, signifying the presence of a positive clone.

Protease activity screening medium was LB medium supplemented with 12.5 mg/L chloramphenicol and with 0.2% (w/v) final concentration of AZCL-casein. Protease screening plates were incubated during 3 weeks at 37°C, in order to observe the eventual apparition of a blue halo around the hit clones.

### 3.4. Secondary screening

The hit clones isolated from primary screening were then assayed to determine their ability to degrade Impranil, using two different solid media.

The solid selective medium contained 3 g/L Impranil as sole carbon source and a minimal medium composed of salts (3.6 g/L Na2HPO4, H2O; 0.62 g/L KH2PO4; 0.11 g/L NaCl; 0.42 g/L NH4Cl), 2 mM MgSO4, 0.03 mM CaCl2, other salts (15 mg/L Na2EDTA, 2H2O; 4.5 mg/L ZnSO4, 7H2O; 0.3 mg/L CoCl2, 6H2O; 1 mg/L MnCl2, 4H2O; 1 mg/L H3BO3; 0.4 mg/L Na2MoO4, 2H2O; 3 mg/L FeSO4, 7H2O; 0.3 mg/L CuSO4, 5H2O), leucine 0.04 g/L and thiamine hypochloride 0.1 g/L and supplemented with 12.5 mg/L chloramphenicol. The assay plates were incubated at 37°C for up to 3 weeks or until growth was observed.

The rich Impranil containing medium was constituted by solid LB medium supplemented with 12.5 mg/L chloramphenicol and with 3 g/L of Impranil in suspension. The assay plates were incubated at 37°C for up to 3 weeks, or until apparition of a degradation halo, signifying the presence of a positive clone.

After clone isolation on solid LB medium and validation of their activity on the Tween20 screening medium, the hit clones obtained after primary screening were also grown in microplates containing 200μL of LB medium supplemented with 12.5 mg/L chloramphenicol for pre-culture, at 37°C overnight. These pre-cultures were used to inoculate cultures in deep well micro-plates containing 1.6 mL of LB liquid medium supplemented with 12.5 mg/L chloramphenicol, which were subsequently incubated overnight at 37°C. Cells were pelleted by centrifugation at 1,760 x g for 30 minutes at 4°C. Each pellet was re-suspended in 250 μL of lysis buffer (100 mM HEPES pH 7.5, 1 mM pefabloc and 666.7 μl/L lysonase), and incubated at 600 rpm (shaking throw 25 mm) for one hour at 37°C in a Multitron Pro shaker (INFORS HT) with a freeze/thaw cycle at -80°C in order to perform cell lysis. Cellular extracts were isolated by centrifugation at 1,760 x g for 30 minutes at 4°C, and stored at 4°C for less than 24 h before use.

Carboxylesterase activity of the cytoplasmic extracts was quantified by monitoring the hydrolysis of *p*NP esters into the corresponding acid and *p*-nitrophenol. Assays were performed in 96-well microtiter plates on *p*NPA, *p*NPB and *p*NPP at 1 mM final concentration. *para*Nitrophenol was used for standard curve. For *p*NPA and *p*NPB, 195 μL of cellular extract were mixed with 5μL of substrate dissolved at 40 mM in 2-methyl-2-butanol (2M2B). For *p*NPP, 175μL of cellular extract were mixed with 25μL of substrate dissolved at 8 mM in isopropanol. Enzymatic activity was determined by following the absorbance increase at 405 nm during 30 min at 30°C, in a microplate spectrophotometer (BioTek™ Eon™ Microplate Spectrophotometers, Colmar, France). A fosmid clone was considered as positive on one of these pNP-substrates if its activity was at least of 5.10^−3^ U/mL of cell extract in these screening conditions.

### 3.5. Sequencing and data analysis

Fosmid DNA of the positive clones was extracted with the NucleoBond Xtra Midi kit from Macherey-Nagel (France) following supplier recommendations. Fosmids were then individually labelled to avoid any further miscontigation [58], and sequenced with the MiSeq technology, at the Genotoul platform (http://get.genotoul.fr/). For each clone, a random selection of reads was performed to obtain a mean depth coverage of 40 x. Read assembly was carried out using Masurca (http://www.genome.umd.edu/masurca.html). Contigs were cleaned from the vector pCC1FOS sequence using Crossmatch (http://bozeman.mbt.washington.edu/phredphrapconsed.html). ORF detection and functional annotation was performed using the RAST software [59]. Sequences sizing between 21 and 38 kbp, which correspond to the largest contig obtained for each clone, were deposited in the European Nucleotide Archive (http://www.ebi.ac.uk/ena) under accession numbers LT674540-LT674547 (Table 1).

The RAST annotation of the carboxylesterase encoding genes was confirmed by BLASTP analysis of the predicted ORFs against the NCBI non-redundant protein sequences database (E-value ≤10^−8^, identity ≥35%, query length coverage ≥50%, the same cut-off parameters as in [40]). Further sequence analysis was performed with other BLASTP analysis against the ESTerases and alpha/beta-Hydrolase Enzymes and Relatives database (ESTHER) [52]. The protein signature was detected using the InterProscan software [60]. Sequence alignments leading to the creation of the phylogenetic tree along with the tree itself were designed using the MEGA6 software [61]. The sequences used were chosen from different publications, cited in the text. A distance matrix was generated from the multiple sequence alignment using the BLOSUM62 amino acid residue substitution matrix. The output result file was subjected to hierarchical clustering using the Ward's method [62]. The multiple alignment of CE_Ubrb sequence with those of its characterized homologs and with members of the lipolytic family IV was generated with Clustal Omega.

Taxonomic assignation of the metagenomic sequences was determined according to PhyloPythias analysis [63] (http://phylopythias.bifo.helmholtz-hzi.de, Model type Generic 2013–800 Genera) of the nucleotide sequence of the largest contigs. Same results were obtained with minimum slice at 3 and 50%.

### 3.6. Characterization of enzymatic activities

#### 3.6.1. Carbamate and polyurethane degradation reactions

The positive clones were grown in tubes containing 3 mL of LB medium supplemented with 12.5 mg/L chloramphenicol for preliminary culture, at 37°C overnight. These cultures were used to inoculate 20 mL cultures grown in LB medium supplemented with 12.5 mg/L chloramphenicol at 37°C, with orbital shaking at 120 rpm (shaking throw 25 mm). After 24 h, cells were harvested by centrifuging for 5 min at 12,857 x g, and re-suspended in activity buffer (PBS pH 7.0) to obtain a final OD_600nm_ of 80. Cell lysis was done using sonication. Cell debris were centrifuged at 12,857 x g for 10 min and the cytoplasmic extracts were filtered with a 0.20 μm Minisart RC4 syringe filter (Sartorius). Quantification of total proteins present in the cytoplasmic extracts was carried out using the Bradford method [64]. The samples were diluted by five, and 20μL of these dilutions were mixed with 200 μL of Bradford reagent. After 20 minutes, the OD was measured at 595 nm. The standard used was BSA.

Overproduction of recombinant proteins was visualized by SDS-PAGE analysis of the cytoplasmic extracts using Any kDTM Mini-PROTEAN_ TGXTM Precast Gel (Bio-Rad). After migration, proteins were stained with the PageBlue Protein Staining Solution (Thermo Scientific) according to the manufacturer’s recommendations. Enzymatic reactions were carried out at 30°C in hemolysis tubes, containing 500 μL of cytoplasmic extract, mixed with 500 μL of the substrate suspended or solubilized in the activity buffer (PBS pH 7.0) to obtain a final concentration of 6 g/L for the Impranil dispersion, or 2 g/L fenoxycarb, fenobucarb or prosulfocarb (for structures see S1 Fig).

The reaction progress was followed by taking samples at 0 and 24h of incubation for LC- MS and Maldi-Toff analyses. Samples were incubated at 95°C for 5 minutes to stop the reaction for the small carbamates. Impranil reactions were directly frozen at—80°C to stop the reaction, in order to avoid polymer chain reorganization that could have impacted the results.

#### 3.6.2. Matrix assisted laser desorption/ionization–time-of-flight (MALDI-TOF) mass spectrometry

Samples were lyophilised before being re-suspended in 400 μL of THF. Samples were then analyzed onto a commercial MALDI-TOF mass spectrometer MALDI-TOF Micro Mx (Waters), Laser N2 (337 nm), acceleration voltage 12 kV, reflectron mode, dithranol matrix and NaI salts, in the linear and positive mode for a range of m/z 200–1,200. Spectra were acquired automatically using a standard procedure at the Service Commun de Spectrométrie de Masse at Université Paul Sabatier in Toulouse, France.

#### 3.6.3. High performance liquid chromatography–mass spectrometry

As in other papers describing carbamate-pesticide degradation [38], we used a LC-18 column and UV detection to quantify carbamate concentration in the samples. LC-18 (octadecyl) are general-purpose hydrophobic alkyl phases that give good peak shape for a variety of compounds, and we checked that the method was quantitative for fenobucarb, fenoxycarb and prosulfocarb by using standard curves prepared from known compound concentrations.

Samples were dissolved in 1.5 volumes of acetonitrile, in order to recover a ratio of 60% acetonitrile, 40% water. Analyses were performed on a HPLC system (Ultimate 3000, Dionex) with in line degasser. LC of small carbamate samples was performed on a SUPELCOSIL™ LC-18 HPLC Column 5 μm particle size (250× 4.6 mm) equipped with a SUPELCOSIL™ LC-18 Supelguard™ Cartridge 5 μm particle size (20 × 4.0 mm) pre-column. The mobile phase (60% acetonitrile-40% water) was pumped at a flow rate of 1 mL/min, and UV detection was carried out at 205 nm, 210 nm and 235 nm for fenobucarb, prosulfocarb and fenoxycarb, respectively.

The LC system was hyphenated with a mass spectrophotometer (MSQ PLUS, Thermo Scientific) operated in positive mode and equipped with an electrospray ionisation (ESI) source for 30 min with a scan time of 0.10 seconds over the range m/z 100–1,500 with nitrogen (N2) as drying gas. A cone of 60 V was used, and the probe temperature was set at 450°C. Fourty percents of the sample went through the RI detector, 60% to the mass spectrometer. The Chromeleon software was used for data acquisition and analysis.

### 3.7. Sub-cloning of the CE_Ubrb encoding gene and validation of carboxylesterase activity

First, the CE-Ubrb encoding gene (accession number SIP63154.1) was PCR-amplified from one single colony of the *Escherichia coli* metagenomic fosmid clone 44I12 by using the CloneAmp^TM^ kit (Gibco BRL) and the primers forward 5’-CACCATGAGCATTCGCGTCATACCG-3’ and reverse 5’-ATCATTTTCGAATCCCTCCGTATTTCTTGC -3’. After PCR (one denaturation step at 98° C for 30 s; 30 cycles composed of a denaturation step at 98°C for 10 sec, an hybridization step at 55°C for 15 sec, an elongation step at 72°C for 30 sec; and a final elongation step at 72°C for 3 min), the PCR product was purified from agarose gel using the GenElute gel extraction kit (Sigma-Aldrich).

The PCR product was then cloned into the pENTR/D-TOPO vector (Invitrogen) according to the manufacturer’s recommendations. After *E*.*coli* OneShot TOP10 transformation, the plasmid was extracted using the QIAGEN Plasmid Purification kit and the construct was checked by Sanger sequencing. By using recombination according to the manufacturer’s recommendations, the CE-Ubrb encoding gene was then cloned into the pET55 destination vector (Novagen), yielding fusion of the recombinant protein to a N-terminal Strep-tag and to a C-terminal (His)_6_ tag.

*E*. *coli* BL21 star (DE3) cells (Invitrogen) harboring the CE-Ubrb encoding plasmid were cultured in Erlenmeyer flasks at 28°C for 24 h in 200 ml ZYM-5052 autoinduction medium [65] supplemented with 100 mg/L ampicillin, inoculated at *A*600 nm 0.05. For comparison, the EPI100 E. *coli* cells transformed with the 44I12 fosmid were cultured in the same conditions, except that the culture medium was LB supplemented with 12.5 mg/L chloramphenicol. For both plasmid and fosmid clones, cells were harvested by centrifuging for 5 min at 12,857 x g, and re-suspended in activity buffer (100 mM Hepes, pH 7.5) to obtain a final OD_600nm_ of 80. Cell lysis was done using sonication. Cell debris were centrifuged at 12,857 x g for 10 min and the cytoplasmic extracts were filtered with a 0.20 μm Minisart RC4 syringe filter (Sartorius). Carboxylesterase activity of the cytoplasmic extracts was quantified by monitoring the hydrolysis of *p*NP-esters into the corresponding acid and *p*-nitrophenol after dilution of the cytoplasmic extracts by 100 times in activity buffer for *p*NPA and *p*NPB, and by 10 times for *p*NPP.

## 4. Conclusion

This work is the first metagenomic study targeting the discovery of fenobucarb and polyurethane degrading activities. One of the carbamate degrading metagenomic clone we isolated produces a new lipolytic family IV carboxylesterase named CE_Ubrb. This original enzyme is very distantly related to all the known carbamate degrading enzymes, which belong to the family VII. The diversity of the sequences we predicted, or proved, to encode carboxylesterases, highlights the interest of complex microbiomes for the mining of promiscuous enzymes of biotechnological interest. Thanks to their flexible substrate specificity, these enzymes could indeed be used to break down man-made contaminating compounds in soil, water and other environments.

## Supporting information

S1 Table Functional profiles of the hit metagenomic clones, and name of the sequenced clones. (DOC)

S1 Fig Structure of Tween 20 and of the carbamates used in this study. (TIF)

S2 Fig A—MALDI-TOF spectrum of Impranil re-suspended in THF. B—MALDI-TOF spectra of the reaction medium containing Impranil and the enzymatic extract of *E*. *coli* strain Epi100 carrying the empty pCC1FOS fosmid at the beginning (B-1) and after 24h (B-2) of reaction.
(PDF)

S3 Fig A—MALDI-TOF spectra of the reaction medium containing poly[4-(2,2-dicyanovinyl)-N-bis(hydroxyethyl)aniline-alt-(4,4’-methylenebis(phenylisocyanate)))]urethane and the enzymatic extract of clone 44I12 at the beginning (A-1) and after 24h (A-2) of reaction; B—MALDI-TOF spectra of the reaction medium containing poly[4-(2,2-dicyanovinyl)-N-bis(hydroxyethyl)aniline-alt-(isophroronediisocyanate)]urethane and the enzymatic extract of clone 44I12 at the beginning (B-1) and after 24h (B-2) of reaction.
(PDF)

S4 Fig HPLC analysis of the reaction medium containing fenobucarb and the enzymatic extract of *E*. *coli* strain Epi100 carrying the empty pCC1FOS fosmid at the beginning (blue) and after 24 h of incubation (red). (TIF)

S5 Fig Graphical representation of the metagenomic sequences of clones 13M17, 25I16, 29D17, 37F15, 39F1, 50E19 and 50I3, and RAST annotation of the identified ORFs. Red arrows: ORFs encoding putative esterases or proteases, based on RAST annotation or on results of BLAST comparison with the NCBI NR database.
(PDF)

S6 Fig SDS-PAGE analysis of the cytoplasmic extracts of the 8 sequenced fosmid clones, highlighting overproduction of proteins of molecular weight similar to that of the putative esterases SIP63261.1 (72,8 kDa, clone 25I16), SIP63154.1 (CE_Ubrb, 32,4 kDa, clone 44I12), SIP63136.1 (72,8 kDa, clone 50E19) and SIP63105.1 (41,4 Kda, clone 50I3), compared to the negative control (*E*. *coli* strain Epi100 carrying the empty pCC1FOS fosmid). (TIF)

S7 Fig SDS-PAGE analysis of the cytoplasmic extracts of the *E*. *coli* clones producing or not CE-Ubrb. A: equivalent volumes of cytoplasmic extracts were deposited in each line; B: equivalent amounts of total cytoplasmic proteins were deposited in each line. Lines 1 and 7: E. coli BL21 star (DE3), lines 2 and 8: *E*. *coli* BL21 star (DE3) transformed with the pET55_CE_Ubrb plasmid, lines 3 and 9: *E*. *coli* BL21 star (DE3) transformed by the pET53_CE_Ubrb plasmid, lines 4 and 10: *E*. *coli* strain Epi100 carrying the 44I12 fosmid, lines 5 and 11: *E*. *coli* strain Epi100 carrying the empty pCC1FOS fosmid.
(TIF)

S8 Fig Multiple alignment of CE_Ubrb sequence with those of its characterized homologs listed in Table 2, and with members of the lipolytic family IV cited in [46]. The amino acids highlighted in red are catalytic amino acids, the green ones the oxyanion hole and the blue ones the GXSXG consensus sequence. “*” is for single, fully conserved residue, “:” shows the conservation between groups of residues bearing strongly similar properties, and “.” between groups of weakly similar properties.
(PDF)
