# Supplementary material for: Discovery of carbamate degrading enzymes by functional metagenomics
Source: PLoS One. 2017 Dec 14;12(12):e0189201. doi: 10.1371/journal.pone.0189201 (PMC5730166; doi:10.1371/journal.pone.0189201)
Supplement: S1 Table — (DOC) [file pone.0189201.s001.doc]

| **Profile number** | **Substrates for discrimination screening** | | | | | | **Number of clones within this profile** | **Sequenced clone number** |
| --- | --- | --- | --- | --- | --- | --- | --- | --- |
|  | Tween 20 | Impranil  (rich medium) | Impranil  (minimal medium) | pNP-A | pNB-B | pNP-P |  |  |
| 1 |  |  |  |  |  |  | 2 | - |
| 2 |  |  |  |  |  |  | 1 | - |
| 3 |  |  |  |  |  |  | 1 | - |
| 4 |  |  |  |  |  |  | 1 | - |
| 5 |  |  |  |  |  |  | 1 | - |
| 6 |  |  |  |  |  |  | 1 | - |
| 7 |  |  |  |  |  |  | 5 | 50I3-37F15 |
| 8 |  |  |  |  |  |  | 1 | - |
| 9 |  |  |  |  |  |  | 4 | 13M17 |
| 10 |  |  |  |  |  |  | 1 | - |
| 11 |  |  |  |  |  |  | 2 | - |
| 12 |  |  |  |  |  |  | 5 | 39F1-25I16-50E19-29D17 |
| 13 |  |  |  |  |  |  | 1 | 44I12 |

**S1 Table**
