# Supplementary figures and images for: Discovery of carbamate degrading enzymes by functional metagenomics
Source: PLoS One. 2017 Dec 14;12(12):e0189201. doi: 10.1371/journal.pone.0189201 (PMC5730166; doi:10.1371/journal.pone.0189201)

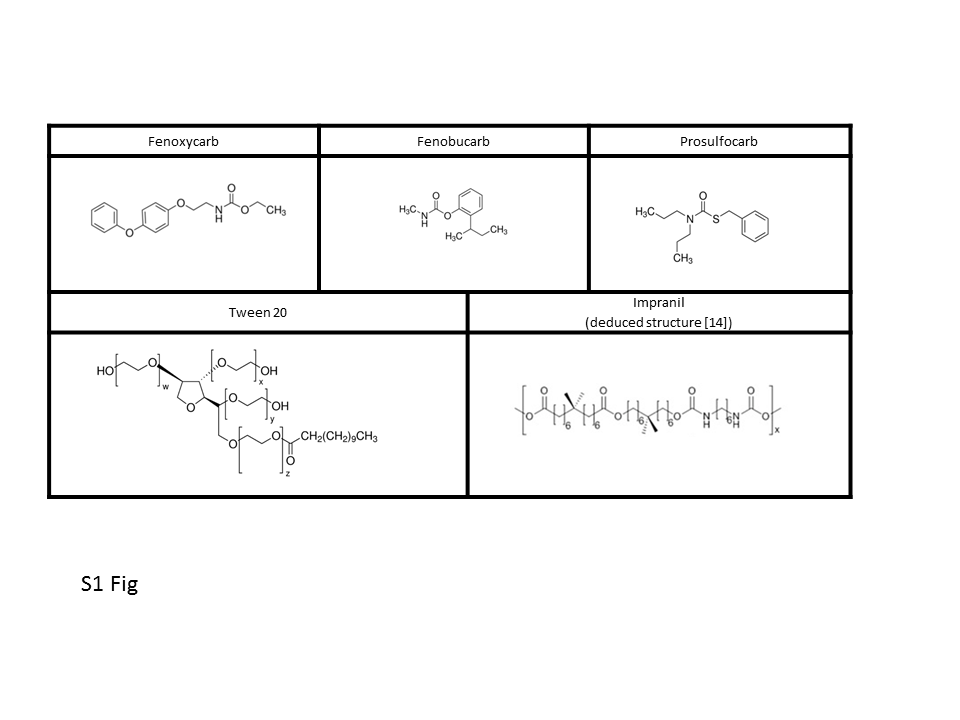

Supplement: S1 Fig — (TIF) [file pone.0189201.s002.tif]

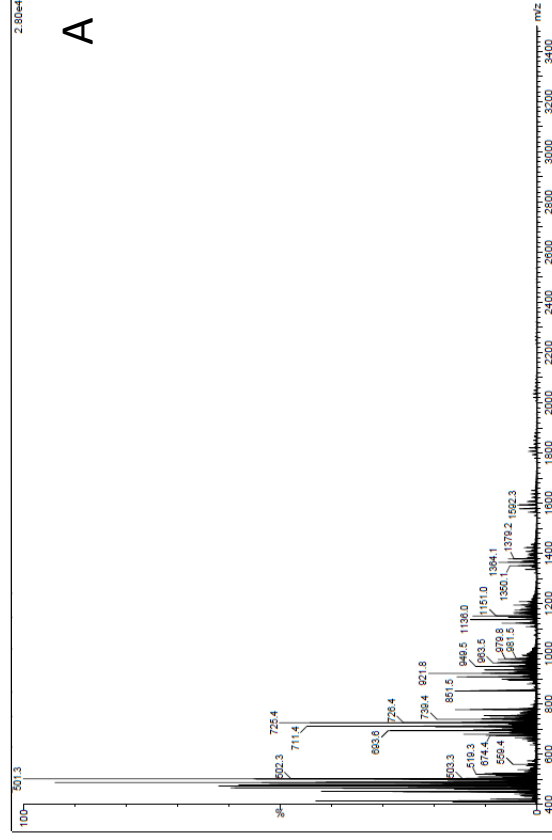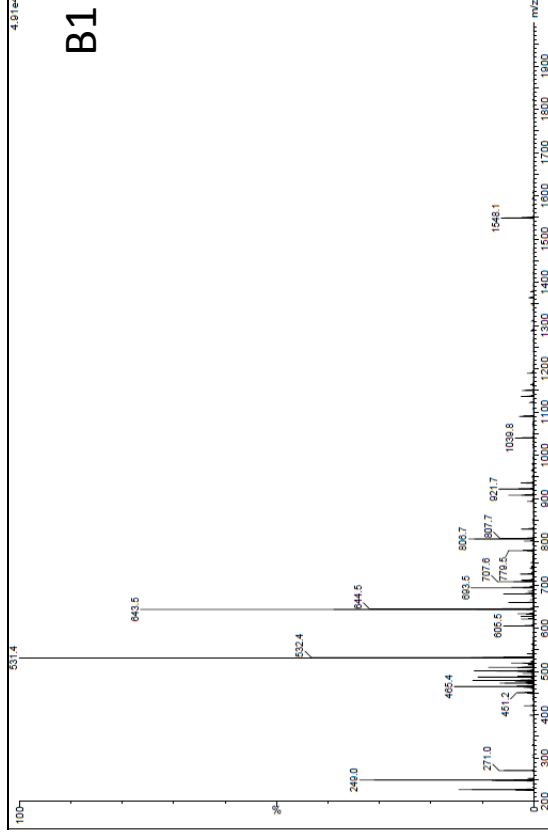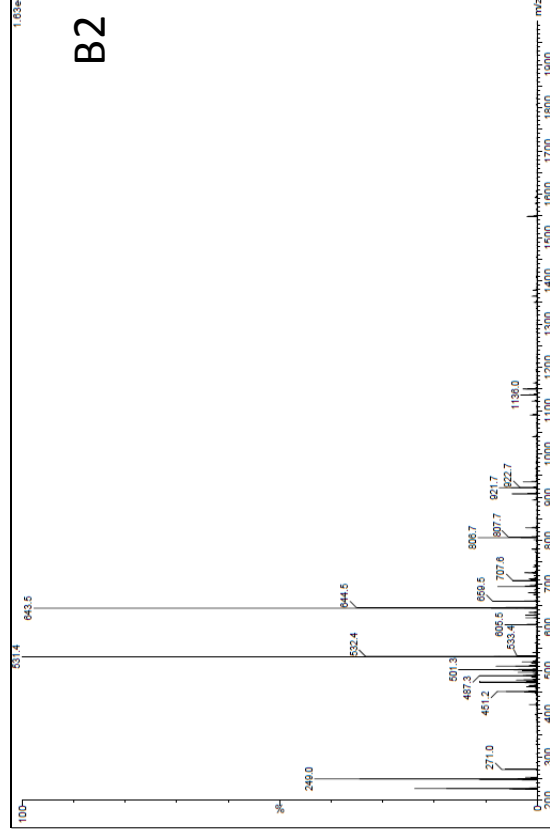

S2 Fig

Supplement: S2 Fig — A—MALDI-TOF spectrum of Impranil re-suspended in THF. B—MALDI-TOF spectra of the reaction medium containing Impranil and the enzymatic extract of E. coli strain Epi100 carrying the empty pCC1FOS fosmid at the beginning (B-1) and after 24h (B-2) of reaction. (PDF) [file pone.0189201.s003.pdf]

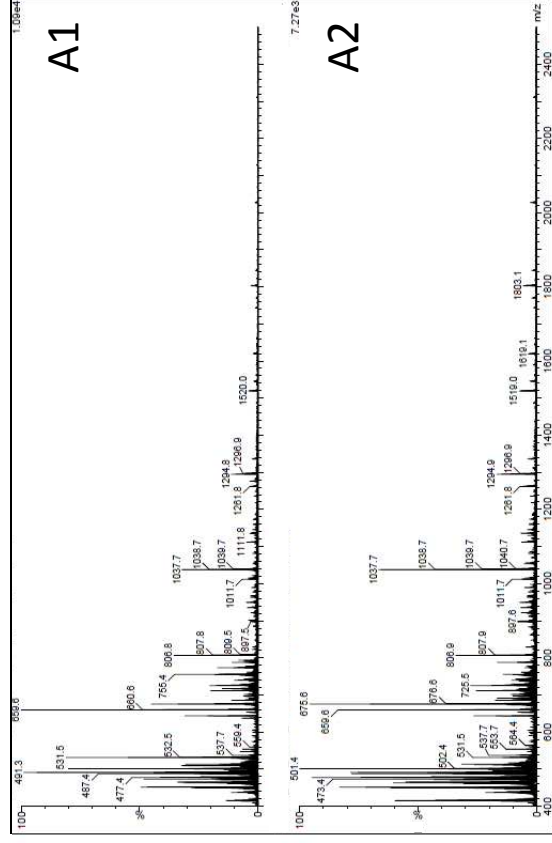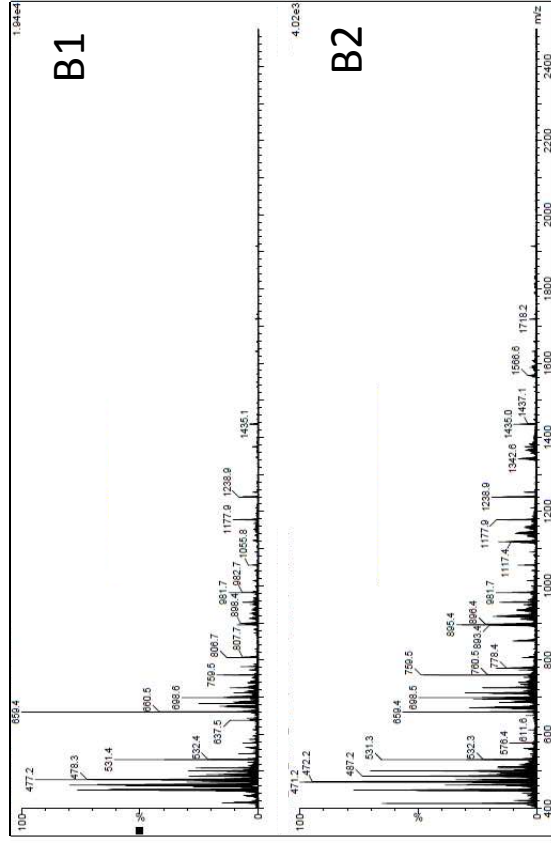

S3 Fig

Supplement: S3 Fig — A—MALDI-TOF spectra of the reaction medium containing poly[4-(2,2-dicyanovinyl)-N-bis(hydroxyethyl)aniline-alt-(4,4’-methylenebis(phenylisocyanate)))]urethane and the enzymatic extract of clone 44I12 at the beginning (A-1) and after 24h (A-2) of reaction; B—MALDI-TOF spectra of the reaction medium containing poly[4-(2,2-dicyanovinyl)-N-bis(hydroxyethyl)aniline-alt-(isophroronediisocyanate)]urethane and the enzymatic extract of clone 44I12 at the beginning (B-1) and after 24h (B-2) of reaction. (PDF) [file pone.0189201.s004.pdf]

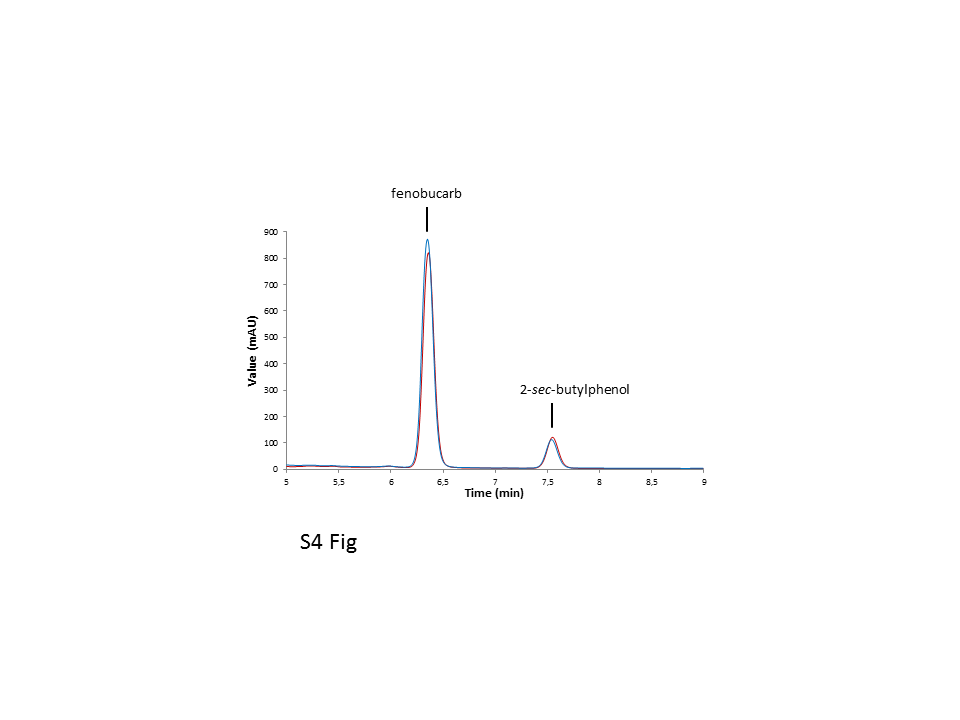

Supplement: S4 Fig — (TIF) [file pone.0189201.s005.tif]

S5 Fig

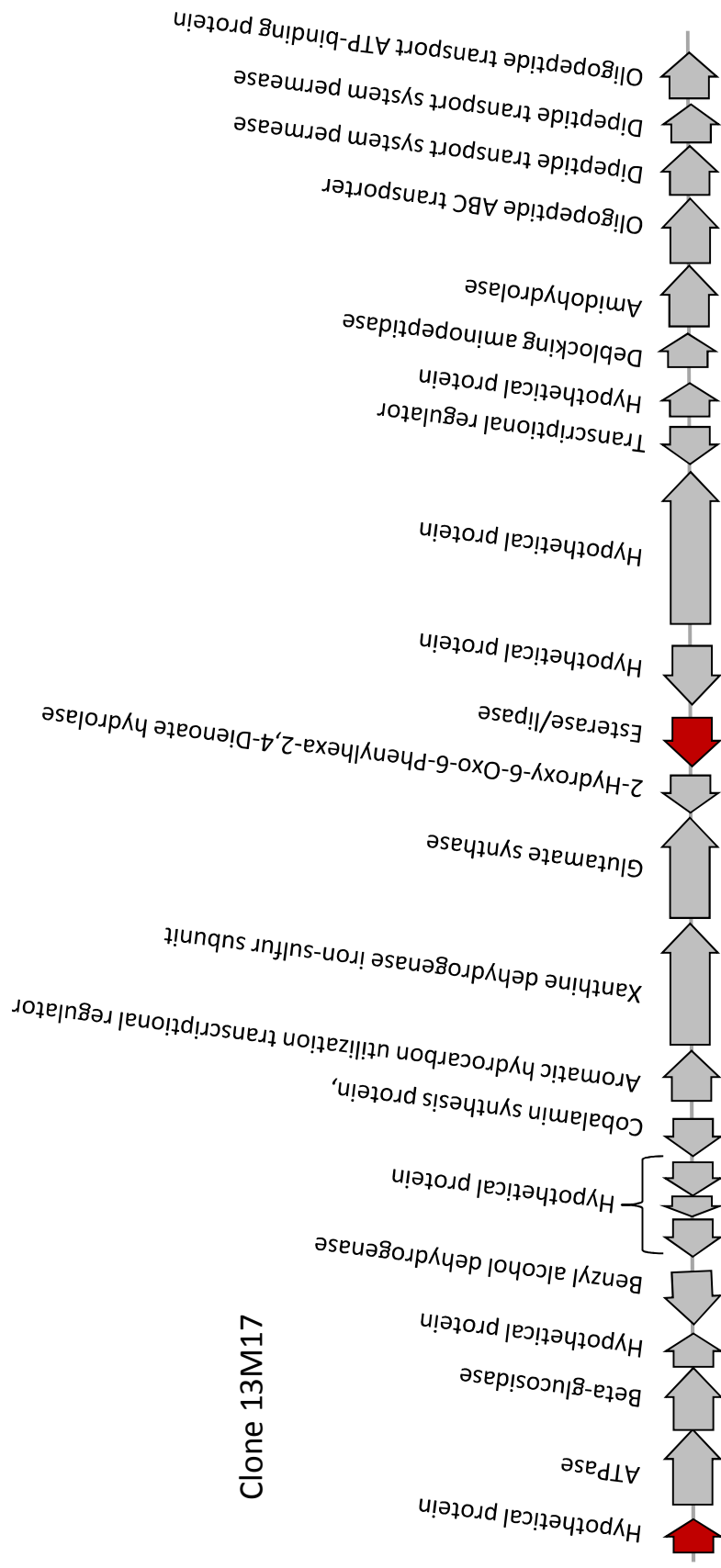

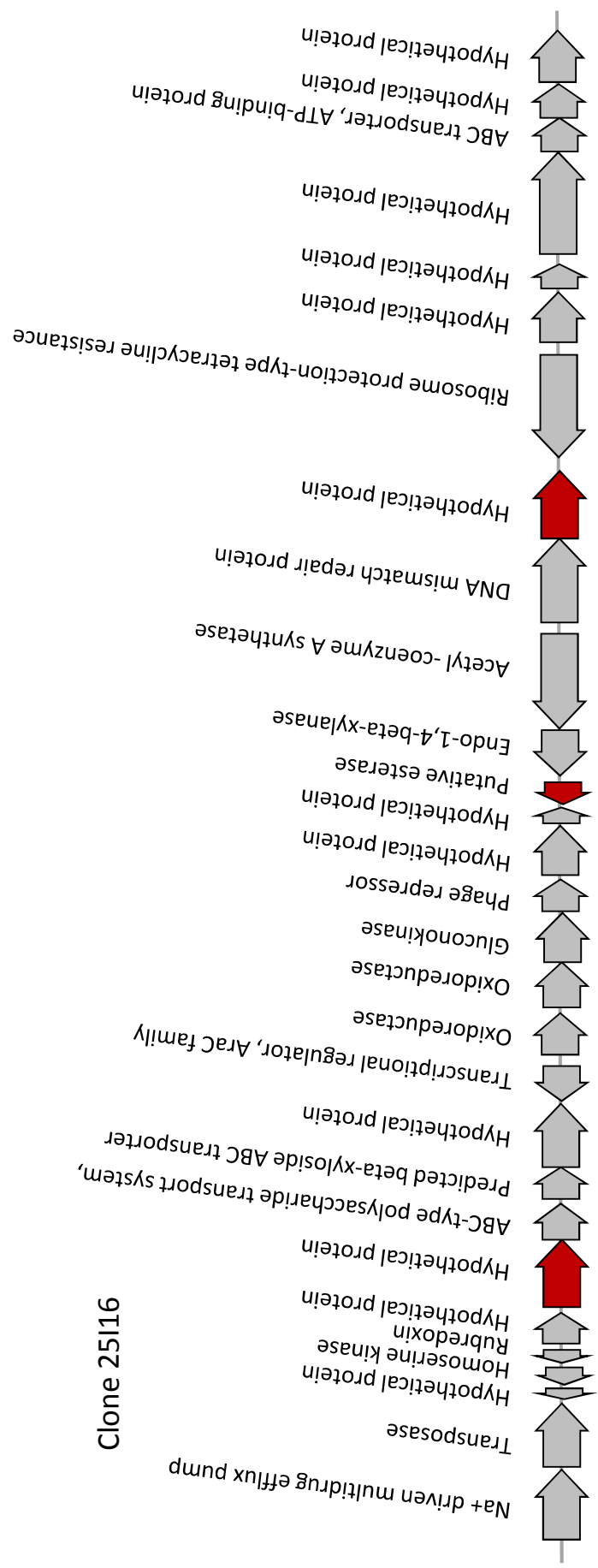

Clone 25116

Clone 29D17

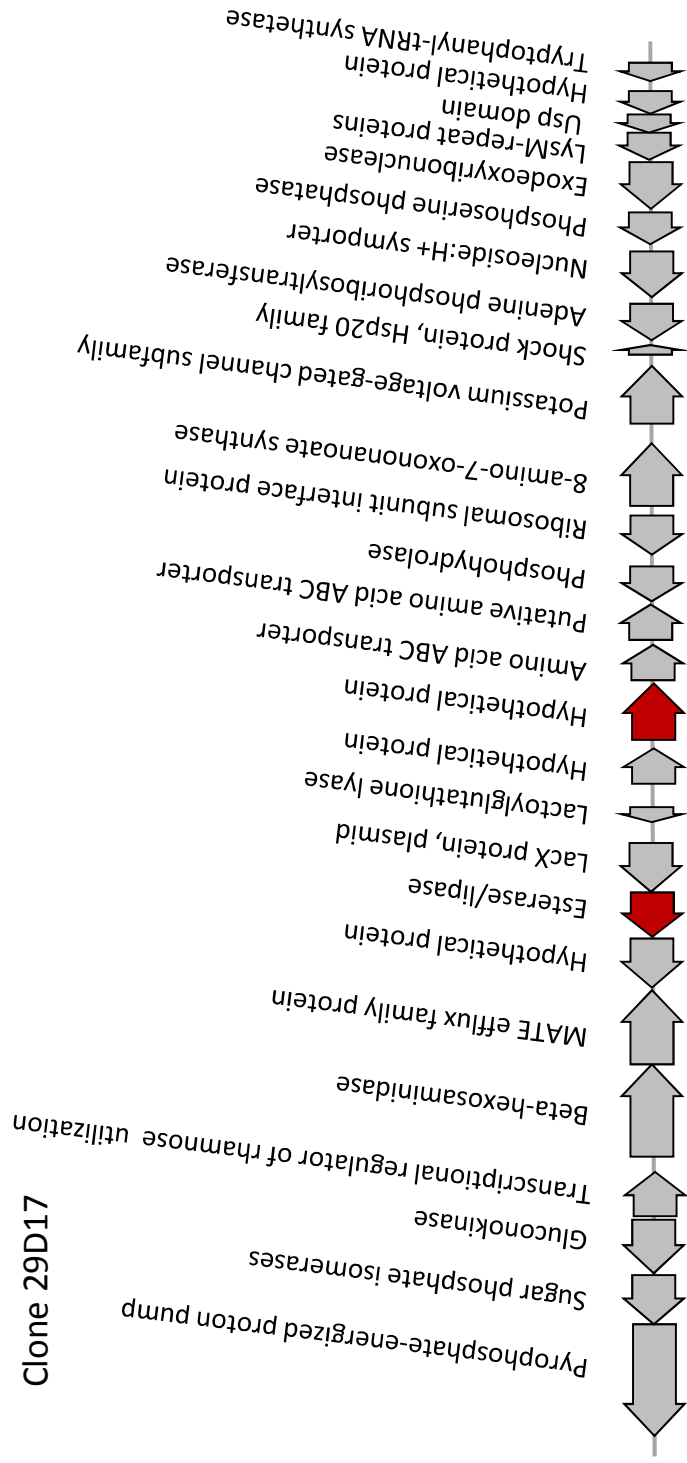

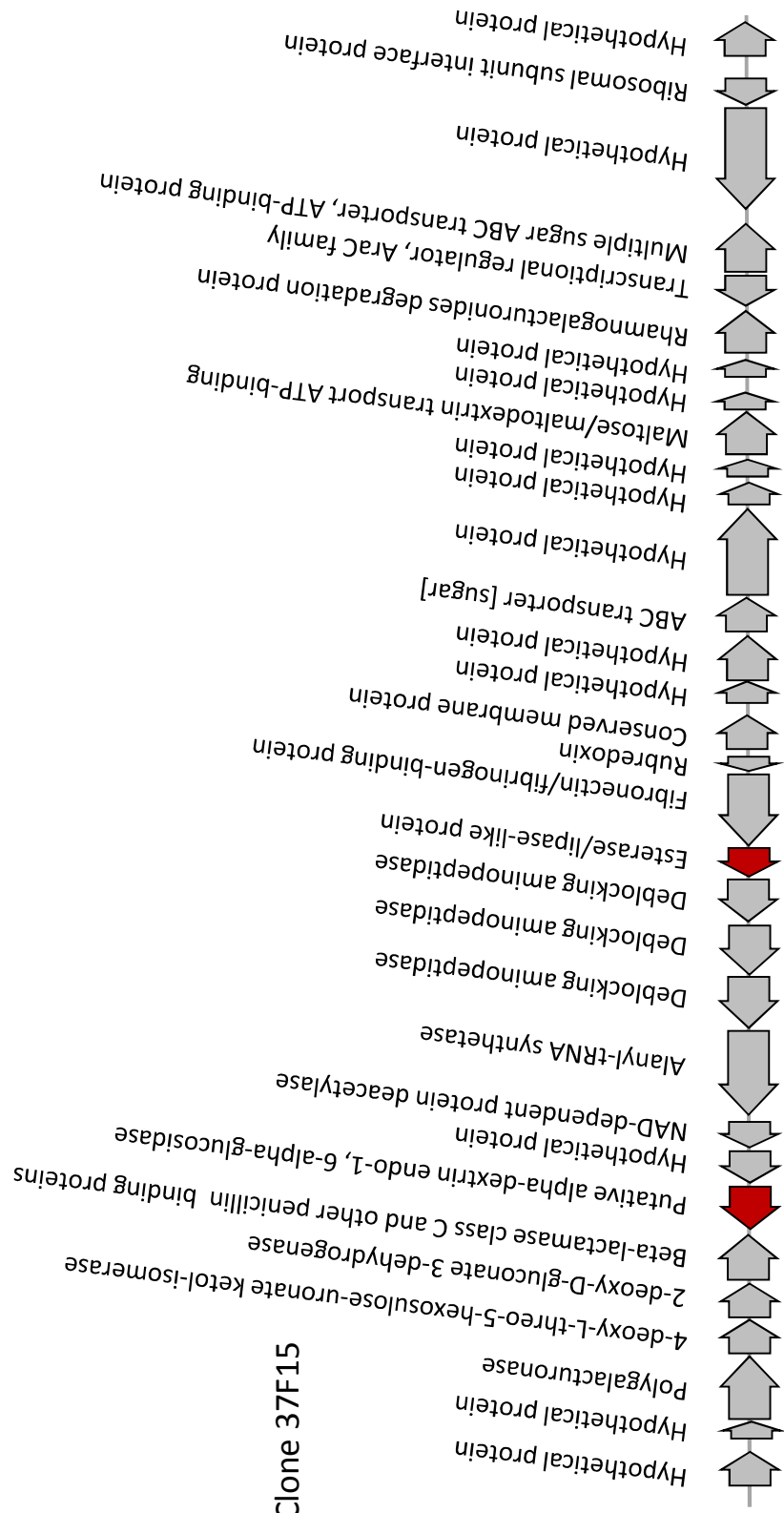

Clone 37F15

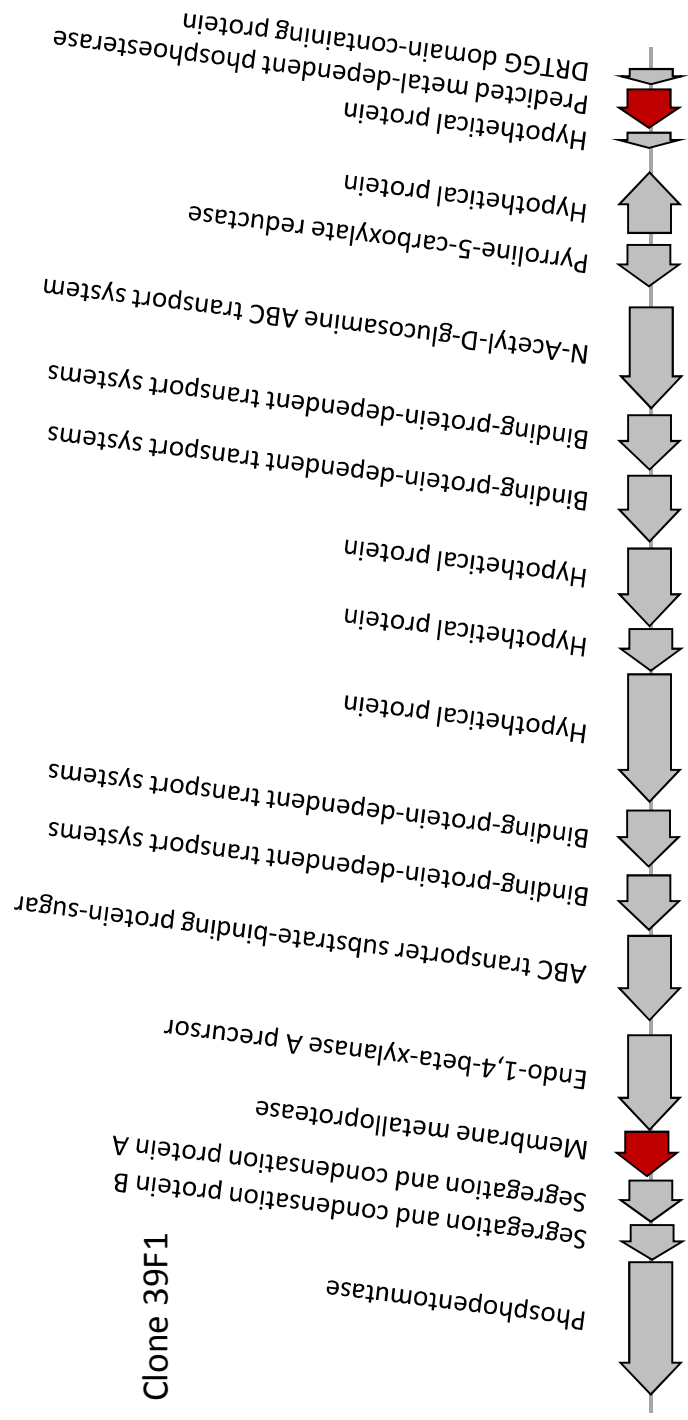

Clone 39F1

Clone 50E19

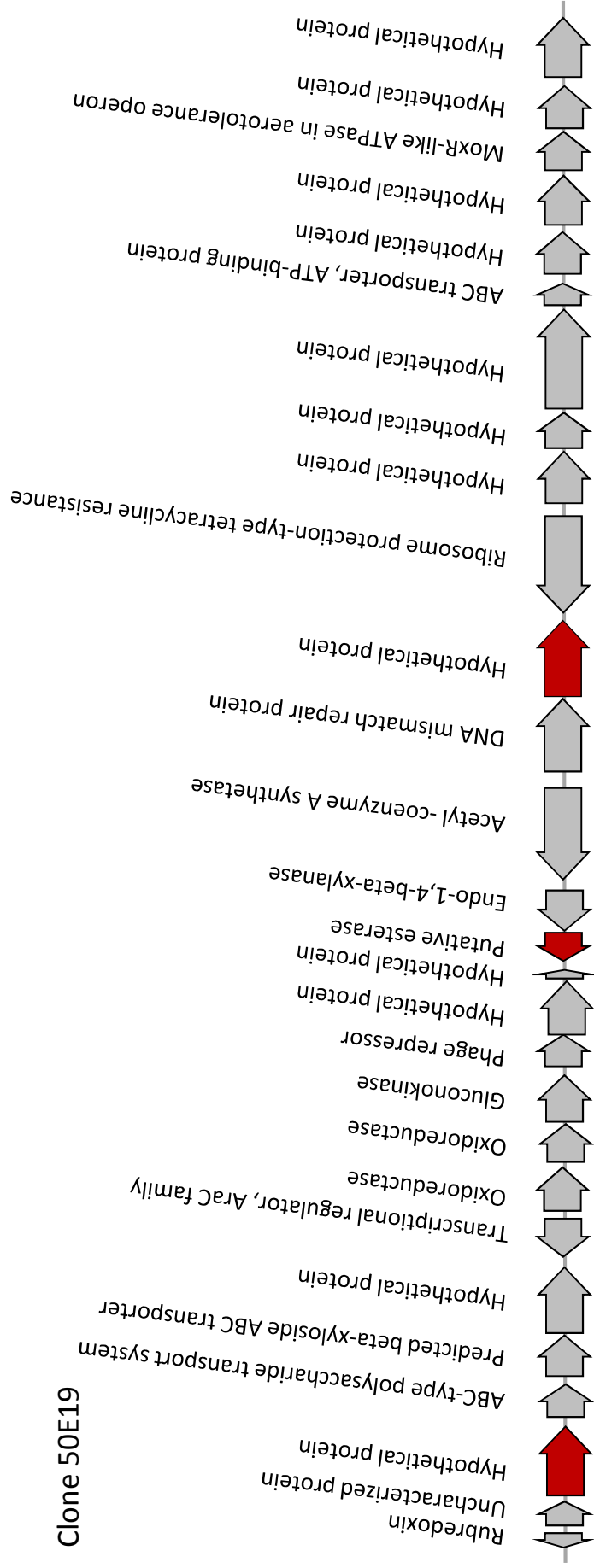

Clone 5013

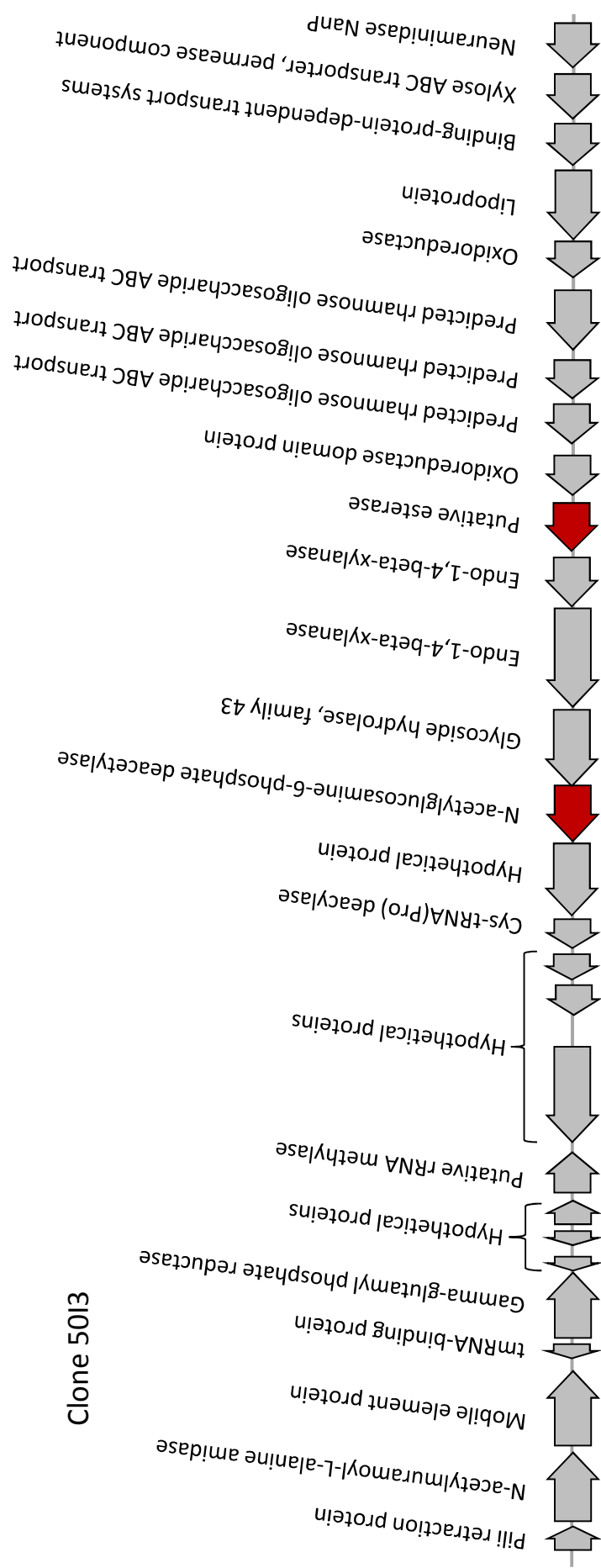

Supplement: S5 Fig — Red arrows: ORFs encoding putative esterases or proteases, based on RAST annotation or on results of BLAST comparison with the NCBI NR database. (PDF) [file pone.0189201.s006.pdf]

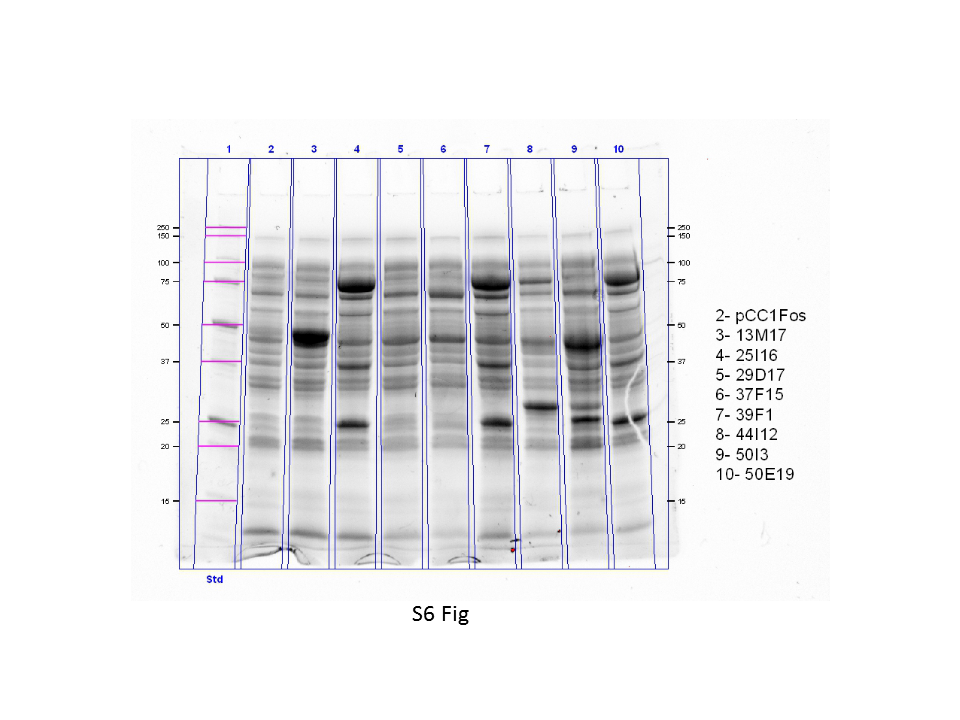

Supplement: S6 Fig — (TIF) [file pone.0189201.s007.tif]

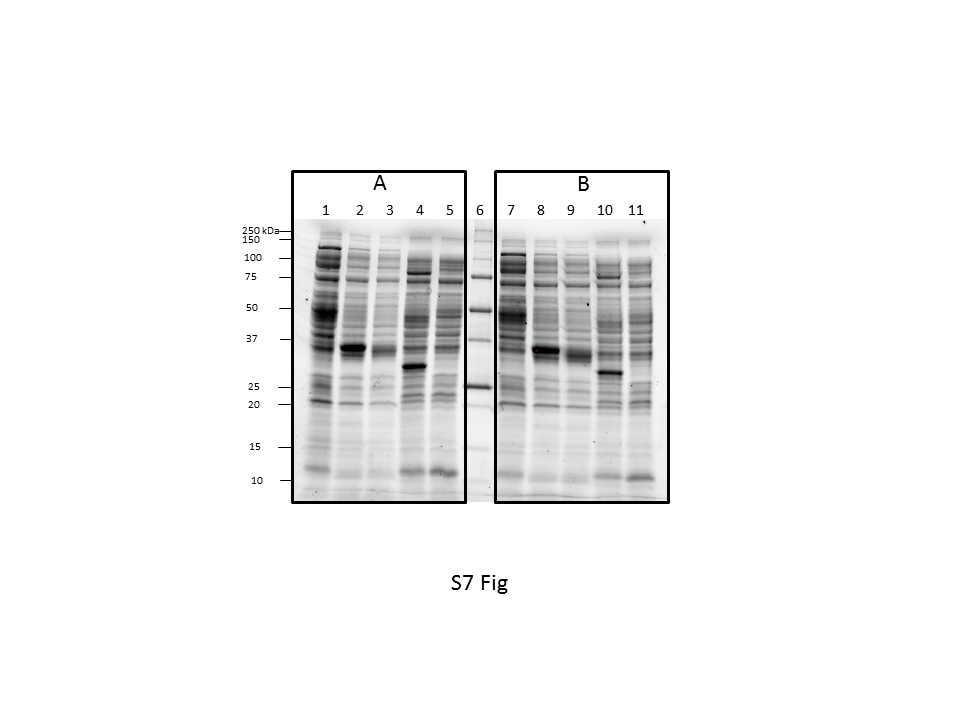

Supplement: S7 Fig — A: equivalent volumes of cytoplasmic extracts were deposited in each line; B: equivalent amounts of total cytoplasmic proteins were deposited in each line. Lines 1 and 7: E. coli BL21 star (DE3), lines 2 and 8: E. coli BL21 star (DE3) transformed with the pET55_CE_Ubrb plasmid, lines 3 and 9: E. coli BL21 star (DE3) transformed by the pET53_CE_Ubrb plasmid, lines 4 and 10: E. coli strain Epi100 carrying the 44I12 fosmid, lines 5 and 11: E. coli strain Epi100 carrying the empty pCC1FOS fosmid. (TIF) [file pone.0189201.s008.tif]
