# Supplementary material for: Discovery of carbamate degrading enzymes by functional metagenomics
Source: PLoS One. 2017 Dec 14;12(12):e0189201. doi: 10.1371/journal.pone.0189201 (PMC5730166; doi:10.1371/journal.pone.0189201)
Supplement: S8 Fig — The amino acids highlighted in red are catalytic amino acids, the green ones the oxyanion hole and the blue ones the GXSXG consensus sequence. “*” is for single, fully conserved residue, “:” shows the conservation between groups of residues bearing strongly similar properties, and “.” between groups of weakly similar properties. (PDF) [file pone.0189201.s009.pdf]

```
NP_005348.2     LELW-PRPQQAP-----RSRSLIVHFHGGGFVAQTSRSHEPYLKSWAQELGAPIISIDYS     684
AAS77245.1      ADIAVPKGT--------GPYPVVVYLHGGGWVAGSPKTHRKLGMHFA-DAGFLTINVDYR     108
ABQ11272.1      ADIAVPKGN--------GPYPVVVYLHGGGWVAGSPKTHRKLGMHFA-DAGFLTINVDYR     108
AAS77242.1      ADIAVPKGN--------GPYPVVVYLHGGGWVAGSPRTHRKLGMHFA-DAGFLTINVDYR     108
AAC41424.1      LRLYTPREASWT-----EPLPLLVYFHGGGFTVGSVDSHDPLCRLLCGQADCMVLSVDYR     163
1EVQ_A          VRXYRPEGV--E-----PPYPALVYYHGGGWVVGDLETHDPVCRVLAKDGRAVVFSVDYR     114
AAC38151.1      ARLYRPLEE--------DNLPLLVFFHGGGFVMGNLDTHDNLCRSLASQTEAVVVSVAYR     114
ACL67843.1      IRVYTPKGD--------TPLPVLVFFHGGGFVIGDLETHDAECRALANAADCIVVSVDYR     114
ACF04196.1      VRIYTPKGE--------GPFPALVYYHGGGWVIGDLETVDVPCRLLTNLANCVVVSVDYR     114
AFG17170.1      MDVFKPAASAKP-----SGI-GVVWMVSGGWYSNHEAINPALSKFFTDRGHTVFQVVHGS     110
AIT56388.1      LDLLLPKKRT-----SDKPLPVVVAIHGGAWLGGDKRQVVGRLIPLVASGEYAGVSVGYR     172
CE_Ubrb         LHIIRPWKRT------TRGYPLVVFIQGSSWTTPDQYWEIPQLSQLAARGFVVATVTHRS     87
EDX41824.1      MSILAPWTQRFPKQYQTEPRPLIVFVQGSSWRTPKMGEEIPQLVQFVQAGYIVATVQHRS     93
                   *          :*  ...:

NP_005348.2     LAPEAPFPRALEECFFAYCWAIKHCALLGSTGERICLAGDSAGGNLCFT--VALRAAAYG     742
AAS77245.1      LAPEHPFPAGLDDCVHAVKWAGENAKRWNGDSSRMAVGGDSAGGNLTAATVTSLSAENYR     168
ABQ11272.1      LAPEHPFPAGLDDCVHAVKWAGENAKRWNGDASRLAVGGDSAGGNLTAATVASLSAENYS     168
AAS77242.1      LAPEHPFPAGLDDCVHAVKWAGENAKRWNGDSSRLAVGGDSAGGNLTAATVTSLSAENYG     168
AAC41424.1      LGPQWRFPTAANDAFDVLHWVFAEAGRLGADPARIAVGGDSAGGTLAAA--CAVEARN-A     220
1EVQ_A          LAPEHKFPAAVEDAYDALQWIAERAADFHLDPARIAVGGDSAGGNLAAV--TSILAKERG     172
AAC38151.1      LAPENHFPAAPLDCYAATCWLVEHAAELGVDGRRLALAGDSAGGNLALA--VSRLAAQRQ     172
ACL67843.1      LAPEHKFPAALDDAFAATEWVASNASAIGADPNRIAVGGDSAGGSLATV--VSQMAKDRG     172
ACF04196.1      LAPEHKFPAAAEDSYAAAKWVAENAASIGVDPNRIAVGGDSAGGNLAAV--VALMARDKR     172
AFG17170.1      Q-PKFTLPEIVQDIHRAMRFIRGHAKEYGVDPERLGICGASAGGHLSLMMAAYGSAGDSN     169
AIT56388.1      LSQDATWPAQIHDCKAAIRWIRGNAKKYNLDPEKIGVIGWSAGGHLVAMLGTSGGVKELE     232
CE_Ubrb         CFEA-KAPAFLTDVKAAIRFLKVKASEYDIDPDRVCAWGTSSGGNAALLVGMTGDDPAFE     146
EDX41824.1      SIDGHPFPAFLQDVKTAIRFLRANAQKYAIDPQQVAIWGTSSGANAAMLVGLTGDDPRYK     153
                    *   : .  : ..     :: * *:*.

NP_005348.2     KSLPPVHIVACALDPML--DDSVMLARRLRNLGQPVTLRVVEDLPHGF-LTLAALCRETR     1037
AAS77245.1      GAMPPSLVICGTADPLL--PESHAIADALKRADIRHEVHIFDDMPHGF-LQMDVLS-GCG     282
ABQ11272.1      GAMPPSLVICGTADPLL--PESYAIADALKRADIRHEVHILEDMPHGF-LQMDVLS-GCG     282
AAS77242.1      GAMPPSLVICGTADPLL--PESHAIADALKRADIRHEVHILEDMPHGF-LQMDVLS-GCG     282
AAC41424.1      RGTCPAWIAVAGYDPLH--DEGVAYAEKLRAAGVAATLADYPGMIHDF-FKLGRFVPAVA     345
1EVQ_A          SGLPPAYIATAQYDPLR--DVGKLYAEALNKAGVKVEIENFEDLIHGF-AQFYSLSPGAT     295
AAC38151.1      ADLPPTTLITAEFDPLR--DEGEAFALRLQQAGVSVRVQRCEGMIHGF-ISMAPFVERAA     293
ACL67843.1      SGLPPALVITAEFDPLR--DDGEAYAARLAEAGVPAKNTRYDGMIHGF-FSMAALLPQAR     293
ACF04196.1      SGLPPALVLTGEFDPLR--DEGEAYAERLKEAGVPVEAKRYDGMIHGF-FWMPGVLEQGR     293
AFG17170.1      TTSPPIFLIHGDADTLVPLQQSQEVINKLEELKVPHKLVVRDGKGHGWPG-LETDLA---     297
AIT56388.1      EDDPPFLLVHGTKDPLVPHQQSAVLRDALRKVGVPAYLITVEEGGH-GGFRNPEIER---     357
CE_Ubrb         RDFPPFLMLHGDADPVVLYEDTERLYRRLVELGYAADLVRVSGAEHEGSFWSETVLE---     273
EDX41824.1      QNYPPFLLFHGDADKVVPYEQMEKMYMRLKDNGNSVEAYRVKGANHERDFWSPTIYN---     277
```

S8 Fig
